# Comparative Evaluation of Four Bacteria-Specific Primer Pairs for 16S rRNA Gene Surveys

**DOI:** 10.3389/fmicb.2017.00494

**Published:** 2017-03-28

**Authors:** Sofie Thijs, Michiel Op De Beeck, Bram Beckers, Sascha Truyens, Vincent Stevens, Jonathan D. Van Hamme, Nele Weyens, Jaco Vangronsveld

**Affiliations:** ^1^Centre for Environmental Sciences, Hasselt UniversityDiepenbeek, Belgium; ^2^Department of Biology, Lund UniversityLund, Sweden; ^3^Department of Biological Sciences, Thompson Rivers UniversityKamloops, BC, Canada

**Keywords:** pyrosequencing, 16S rRNA gene sequence primers, microbial communities, soil, explosives contamination

## Abstract

Bacterial taxonomic community analyses using PCR-amplification of the 16S rRNA gene and high-throughput sequencing has become a cornerstone in microbiology research. To reliably detect the members, or operational taxonomic units (OTUs), that make up bacterial communities, taxonomic surveys rely on the use of the most informative PCR primers to amplify the broad range of phylotypes present in up-to-date reference databases. However, primers specific for the domain Bacteria were often developed some time ago against database versions that are now out of date. Here we evaluated the performance of four bacterial primers for characterizing complex microbial communities in explosives contaminated and non-contaminated forest soil and by *in silico* evaluation against the current SILVA123 database. Primer pair 341f/785r produced the highest number of bacterial OTUs, phylogenetic richness, Shannon diversity, low non-specificity and most reproducible results, followed by 967f/1391r and 799f/1193r. Primer pair 68f/518r showed overall low coverage and a bias toward Alphaproteobacteria. *In silico*, primer pair 341f/785r showed the highest coverage of the domain Bacteria (96.1%) with no obvious bias toward the majority of bacterial species. This suggests the high utility of primer pair 341f/785r for soil and plant-associated bacterial microbiome studies.

## Introduction

High-throughput amplicon sequencing of taxonomic marker genes has become a widely used technique to obtain information on the microbial community composition, diversity, and richness at high resolution in diverse environmental samples (Caporaso et al., 2011; Yergeau et al., 2012a,b). This information has led to significantly increased understanding of the effect of natural or human-induced disturbance on soil and plant-associated microbial communities (Hart et al., 2015; Yergeau et al., 2015; Beckers et al., 2016). While there is no doubt that short-read sequencing technologies have opened up a new dimension of microbial biodiversity research, the technologies also add complexity to the experimental study design. One of the most critical steps for accurate prokaryotic rRNA gene amplicon analyses is the choice of primers.

Primers designed for 16S rDNA usually target a single stretch of the hypervariable regions of the 16S rRNA gene, allowing for species-level taxonomic classification in some cases (Baker et al., 2003; Lundberg et al., 2013). Selection of the V-region targeted and associated primer pairs should therefore be done with care to ensure coverage of relevant microbial taxa and accurate representation of the microbiota profiles in microbiome analyses (Berry et al., 2011; Soergel et al., 2012; Ghyselinck et al., 2013; Walters et al., 2016). Using suboptimal primer pairs can lead to under-representation of certain taxa or selection against single species which can lead to questionable biological conclusions. Moreover, amplification of non-target sequences can consume unnecessary sequencing resources.

Recent attempts have been concerned with optimizing the prokaryotic primer set 515f/806r to target both Bacteria and Archaea (Apprill et al., 2015; Parada et al., 2015; Walters et al., 2016). While this primer pair, used traditionally by the Earth Microbiome Project (Gilbert et al., 2014), is indisputably important in soil ecology studies, it is also crucially important to continue to test and validate other primer sets ensuring that we can continue to uncover the wealth of information that is present in microbial communities. In some studies, it is even more desirable to focus on bacteria alone to centralize the effort and expenses to optimally address the research question. For example, in the field of bioremediation, several reports focus on re-structuring bacterial communities to improve the efficiency of organic contaminant degradation (Bell et al., 2013a,b; Thijs et al., 2016), monitor bacterial community structure in response to pollution (Truyens et al., 2013; Bell et al., 2015), or in response to agricultural management (Jenkins et al., 2016), plant genetic modification (Beckers et al., 2016), and to study the connections between plant-associated bacteria and human/animal microflora (Berg et al., 2015; Mahnert et al., 2015). For all these studies, next to universal prokaryotic primers, adequate bacteria-specific primers that are carefully selected in terms of coverage, reproducibility, exclusion of the amplification of host-organelles, and comparability between studies is needed.

Plant-associated microbiome studies often rely on the use of primers tested against databases from some years ago. In an extensive study by Klindworth et al. (2013), 175 broad-range 16S rRNA gene primers and 512 primer pairs were tested *in silico* against the 376 437 16S/18S rRNA gene sequences in the SILVA v108 database for amplification of bacteria and archaea sequences. However, since 2013, an additional 222,034 sequences have been added to the SILVA database, including new microbial lineages with potentially poor or sub-optimal binding of existing PCR-primers (Quast et al., 2013; Hug et al., 2016). It is therefore important that in light of the ongoing refinements to microbial phylogeny and taxonomy and the increased use of high-throughput sequencing technologies, up-to-date and broadly applicable bacterial primers are re-evaluated to assure the readiness for rational exploration of bacterial diversity and community structure.

Bacteria play an important role in organic compound transformation including the breakdown and detoxification of organic contaminants. Soil bacterial communities have therefore been the subject of bioremediation studies aiming to optimize bacterial community function for accelerating biodegradation and soil remediation (Yang et al., 2012; Yergeau et al., 2012a, 2015). Primer pair 341f/785r used by Klindworth et al. (2013) has been used on a variety of sample types, from marine samples to human samples (Hiergeist et al., 2016), but until now it has not been thoroughly tested on soil. Other primer pairs targeting the V3–V4 region, namely 341f/533r (Yergeau et al., 2014) and 343f/806r (Yergeau et al., 2015) have shown to amplify diverse bacterial communities in the rhizosphere of *Salix purpurea*, suggesting the utility of V3–V4 primer pairs for plant microbiome research.

When amplifying regions of the 16S rRNA gene from soil and plant-associated samples, it is also important to reduce the amplification of non-target DNA-sequences such as those co-extracted from chloroplasts and mitochondria. To account for this, a chloroplast mismatch primer such as 799f (Chelius and Triplett, 2001) may be included. Alternatively, the use of peptide-nucleic acid (PNA) PCR-clamps has been suggested (Lundberg et al., 2013). However, the effectiveness of both approaches to reduce host-organelle amplification depends largely on the plant species. In addition, modifications in the primer pair to reduce chloroplast amplification can also introduce new biases against bacterial groups which are abundant in soil (Lundberg et al., 2012). It is therefore important to carefully compare the performance of primer pairs in terms of taxonomic coverage and non-target amplification to gain the most out of the sequencing effort.

The objective of this study was to test the experimental applicability of four bacteria-specific primer pairs in order to maximize amplification efficiency, taxonomic coverage and target specificity. In contrast to other studies focusing on the evaluation of universal 16S rRNA gene primers, we focus here on the evaluation and observation of the bacterial community in more detail. Inspired by the study of Klindworth et al. (2013), we included primer pair 341f/785r in addition to three other common primer sets spanning different regions of the 16S rRNA gene. The primer pairs were tested empirically on a 2,4,6-trinitrotoluene (TNT)-contaminated soil, a non-contaminated forest bulk soil and *Acer pseudoplatanus* rhizosphere soil, habitats known to harbor diverse sets of bacteria. Additionally, the primer pairs were evaluated *in silico* against the SILVA v123 database, the latest version available at time of writing. The results showed significant differences in taxonomic coverage, diversity, reproducibility, exclusion of chloroplast and ability to discriminate between polluted and non-polluted soils, depending on the primer pair used. We found that 341f/785r detected the highest bacterial diversity, broadest taxonomic coverage, and provided the most reproducible results. Additionally, TNT was found to significantly alter soil bacterial community structure and change the relative abundance of several OTUs.

## Materials and methods

### Study site and soil sampling

Soil samples were collected from a military TNT-production facility in Zwijndrecht, Belgium (51°11′40.0″N; 4°19′29.6″E) on July 8th 2013. The soil was a loamy sand with a moderately thick litter layer and humus with undecomposed and partly degraded organic matter. The TNT-contaminated soil had an average pH of 6.1, moisture content of 66.2%, conductivity of 340 μS per cm, cation exchange capacity of 9.2 Meq per 100 g dry weight using the ammonium acetate method at pH 7 (Chapman, 1965) and 3.1 mg organic labile carbon per kg dry weight soil measured using the permanganate oxidation method (Culman et al., 2012). The non-contaminated soil, a few meters away from the TNT-contaminated location, had an average pH of 7.5, moisture content of 24%, conductivity of 418 μS per cm, cation exchange capacity of 9.5 Meq per 100 g dry weight and 1.6 mg organic labile carbon per kg dry weight soil. Extraction of explosives from soil was performed according to EPA-method 8330. HPLC analyses were performed on an Agilent 1100 system (Agilent, Santa Clara, CA) with Chromosphere C18 reverse phase column (5 μm, 250 × 4.6 mm). TNT-concentrations were calculated using analytical standards (AccuStandard Explosives Inc., New Haven, USA). The concentration of explosives in the contaminated soil was on average 48,835 mg TNT per kg DW soil, 190 mg 1,3,5-trinitrobenzene, 73 mg nitrobenzene, 1,267 mg aminodinitrotoluene, and 182 mg dinitrotoluene per kg DW soil. No TNT or related nitroaromatics were detected in the non-contaminated bulk soil or rhizosphere (<0.01 ppm detection limit).

One hundred gram soil samples were collected from the top 5–10 cm after manually removing the fallen debris and litter. Three replicate soil samples were collected within 20 cm and pooled together to make one sample. Rhizosphere samples were collected from the roots of *A. pseudoplatanus* trees according to standard procedures. All samples were kept at 4°C during transport to laboratory. In the laboratory, bulk soil was sieved using a two mm sieve to homogenize the samples. Rhizosphere soil was obtained by shaking and vigorously vortexing the roots (10 min at max. speed) in sterile P-buffer (per l: 2.36 g Na_2_HPO_4_; 1.80 g NaH_2_PO_4_, 85.0 g NaCl and 200 μl Tween 80; pH 6.8). The tubes were subjected to centrifugation (2,500 g, 20 min), and the resulting pellet was kept as the rhizosphere soil. All soil samples were flash-frozen in liquid nitrogen and stored at −80°C until DNA was extracted.

### DNA extraction, PCR amplification, and pyrosequencing

Approximately 400 mg of soil was used for each DNA extraction. DNA was extracted in triplicate from each pooled sample using a PowerSoil DNA Isolation Kit according to the manufacturer's protocol (MoBio, Carlsbad, CA, USA). This resulted in three replicates for each of six pooled soil samples. Subsequently, amplicon libraries were created using barcode-tagged primers for the primer pairs 68f/518r, 341f/785r, 799f/1193r, 967f/1391r generating amplicons of ~400 bp (Table 1). Both forward and reverse primers were synthesized with the Roche 454 pyrosequencing adaptors (Lib-L A and B) and sample-specific 10 bp multiplex identifier (MID) to allow for sample binning after sequencing (Roche Applied Science, Mannheim, Germany). The forward fusion primer design was 5′ CCATCTCATCCCTGCGTGTCTCCGACTCAGNNNNNNNNNN-forward primer 3′, and the reverse primer design was 5′ CCTATCCCCTGTGTGCCTTGGCAGTCTCAG-reverse primer 3′. A two-round amplification process was used to amplify the DNA samples to reduce dimer formation that is more frequent when using long fusion primers directly on complex genomic DNA-templates. In addition, the first PCR produces homogenous sized amplicons resulting in a more efficient second PCR using the fusion primers (Wu et al., 2010; Berry et al., 2011). The DNA-samples were amplified using a Techne TC-5000 thermocycler (Bibby Scientific Limited, Staffordshire, UK) under the following conditions: initial denaturation at 95°C for 2 min, followed by 25 cycles (1st PCR) or 10 cycles (2nd PCR) of denaturation at 95°C for 30 s, annealing at 53°C for 40 s and extension at 72°C for 1 min, with a final extension performed at 72°C for 10 min. Reactions were carried out in 25 μl reaction volumes using the FastStart High Fidelity PCR System (Roche Applied Science, Mannheim, Germany). Each reaction contained 2.75 ml FastStart 10 × reaction buffer, 1.8 mM MgCl_2_, 0.2 mM dNTP mix, 0.3 mM of each primer, 1.25 U FastStart HiFi polymerase and 5 ng of template DNA for the first round of PCR (as measured using a Nanodrop spectrophotometer). Amplified DNA from the first PCR was purified from 1.5% agarose gel using a QIAquick gel extraction kit (Qiagen, Venlo, Netherlands), and 1 μl of the purified PCR-products was used for the second PCR with the MID-tagged fusion primers using 10 PCR cycles instead of 25. The amplified DNA was cleared from PCR primers and primer dimers using the QIAquick PCR purification kit (Qiagen, Venlo, Netherlands). Finally, purified DNA was quantitated using the Quant-iT PicoGreen dsDNA assay kit (Life Technologies Europe, Gent, Belgium) and a Fluostar Omega plate reader (BMG Labtech, Ortenberg, Germany) prior to pooling amplicons in equimolar concentrations in 100 μl TE buffer. Correct amplicon size and integrity were checked by analysing 1 μl of the library on a DNA-chip (DNA 1000 kit, Agilent Technologies, Diegem, Belgium) on a 2100 Bioanalyser (Agilent Mannheim, Germany) according to the manufacturer's instructions. The resulting quality checked amplicon pool, containing all 24 samples were sequenced using a Roche Genome Sequencer FLX System at LGC Genomics (Berlin, Germany).

**Table 1:** **Primers used in the current study**.

**Primer pair^a^**	**Sequence (5′-3′)**	**Position in *E. coli*^a^**	**V-regions**	**Amplicon size**	**Domain coverage (%)^b^**			**References**
					**Bact**.	**Archaea**	**Eukaryota**	
68f	TNANACATGCAAGTCGRRCG	49–68	V1–V3	450	78.59	1.37	0.001	McAllister et al., 2011
518r	WTTACCGCGGCTGCTGG	518–534						Lee et al., 2010
341f	CCTACGGGNGGCWGCAG	341–357	V3–V4	444	96.51	82.96	0.14	Klindworth et al., 2013
785r	GACTACHVGGGTATCTAATCC	785–805						
799f	AACMGGATTAGATACCCKG	781–799	V5–V7	394	85.55	0	0	Chelius and Triplett, 2001
1193r	ACGTCATCCCCACCTTCC	1177–1194						Bodenhausen et al., 2013
967f	CAACGCGAAGAACCTTACC	967–985	V6–V8	424	66.54	0.007	0.03	Sogin et al., 2006
1391r	GACGGGCGGTGWGTRCA	1391–1407						Walker and Pace, 2007

*Primer sequence, 16S rRNA gene variable region, amplicon size, domain coverage rate and literature reference*.

a *Primer position according to E. coli numbering (Baker et al., 2003) umulative-sum-scal*.

b *Primer pair coverage was calculated using PrimerProspector 1.0.1 according to the non-redundant SILVA SSU r123 database and the weighted score method (Walters et al., 2011)*.

### Bioinformatics processing

The raw sequence data (SFF-files) were pre-processed in QIIME v1.9.1 (Caporaso et al., 2010b) using a custom pipeline and the Flemish Supercomputer Centre (VSC). Sequences were trimmed to a length of 400 bp and were only included in the analyses that matched perfectly to the barcode, had <6 homopolymers, no ambiguous bases, a minimal quality score of 25 over a window of 50 bp, and <2 mismatches in the primers (in case of degenerate primers). Next, the sequences were checked for chimeras using UCHIME v4.2.40 (Edgar et al., 2011) with usearch61_minh set to 1 to reduce the number of false-positive chimeras. After removing the chimeric sequences, the sequences that passed pre-processing were clustered in operational taxonomic units (OTUs) at 97% similarity using Uclust v1.2.22 and closed-reference OTU-picking in QIIME (Caporaso et al., 2010b). Taxonomy was assigned using the SILVA v123 database (Quast et al., 2013). We have chosen SILVA as we wanted to confirm that the primer pairs did not amplify eukaryotes, which are represented in the SILVA database and not in the Greengenes databases. The OTU-table was subsequently depleted of non-target sequences (plant-organelles, Eukaryota, Archaea). The final representative sequence set was aligned using PYNAST v0.1 (Caporaso et al., 2010a) and the core alignment of the SILVA database release 123. Finally, alignments were filtered using the PH Lane mask (Caporaso et al., 2010a) and phylogenetic trees were constructed from the filtered alignments using FastTree v2.1.3 (Price et al., 2009).

Rarefaction curves were constructed for each sample individually as well as per primer pair and soil type using QIIME (Caporaso et al., 2010b). Intra-sample diversity measures including phylogenetic richness, observed species, Shannon diversity and Inverse Simpson were calculated in QIIME on OTU tables that were subsampled to 1,000 reads without replacement as in the hypergeometric model (Colwell et al., 2012) and on non-normalized OTU-tables. Taxonomic plots of the relative abundance data were visualized using Explicet v2.10.5 (Robertson et al., 2013). MetagenomeSeq's cumulative-sum-scaling (CSS; Paulson et al., 2013) was used as normalization method prior to β-diversity analyses. Additionally, global singletons were filtered from the OTU-tables using the filter_otus_from_otu_table script in QIIME. Principal-coordinate analysis (PCoA) was performed on the weighted UniFrac matrices (Lozupone and Knight, 2007) to assess the clustering patterns observed for the samples amplified with the different primers. Permutational multivariate analysis was used to identify whether the observed clustering was significant based on the ANOSIM function in QIIME (Caporaso et al., 2010b). Hierarchical trees were generated from the weighted UniFrac distance matrix and Bray-Curtis dissimilarity matrix using Unweighted Pair Group Method with Arithmetic Mean (UPGMA; Felsenstein, 2003) and Jackknife tree node support.

### Differential abundance testing

To identify differently abundant OTUs between the primer sets or between the soil types, non-parametric and parametric tests were employed. For the non-parametric test, Linear Discriminant Analysis with Effect Size (LEfSe) was used on the relative abundance OTU table (Segata et al., 2011). In brief, Kruskal–Wallis (KW) sum-rank test was run at *p* < 0.05 and in the second step, the Wilcoxon rank-sum test was run at *p* < 0.05. Then Linear Discriminant Analysis (LDA) was run at different effect sizes to detect the most important differently abundant clades. The software package STAMP 8.30 was also used to detect differentially abundant OTUs between two sample groups using the Welch's *t*-test at *p* < 0.05 with 10,000 bootstraps (Parks et al., 2014). For the parametric approach, the zero-inflated Gaussian model (fitZIG) was fitted using the package MetagenomeSeq in R 3.3.0 (Paulson et al., 2013). From the normalized data, a dual heatmap-dendrogram of significant OTUs was constructed based on the log2-transformed abundance values and Bray-Curtis clustering algorithm.

Other statistical analyses were performed in R version 3.3.0 (The R Foundation for Statistical Computing, Vienna, Austria), Past 3.x (http://folk.uio.no/ohammer/past/), and Sigmaplot 11.0 (Systat Software, California, USA). Normal distributions of the residuals of models were checked with the Shapiro-Wilk test, while homoscedasticity of variances was analyzed using the Bartlett's test. Depending on the distribution of the estimated parameters, either ANOVA or the Kruskal–Wallis Rank Sum Test was used to check for significant differences in variances of parameters. Pearson rank sum correlations were run on the biological replicates using Sigmaplot 11.0. Graphical tree representations of differentially expressed clades were generated using GraPhIan (http://huttenhower.sph.harvard.edu/graphlan). To visualize the shared and unique OTUs between samples, the tool Venny 2.1.0 was used (Oliveros, 2007).

### Quantitative real-time PCR

To test the primer pair amplification efficiency on heterogeneous environmental DNA, a two-fold dilution series (1:1–1:32) was made from 12 DNA samples (ranging from 5 to 0.15 ng per μl), and amplified with qPCR. Amplification was performed in optical 96 well plates using a 7500 Fast Real-Time PCR System (Applied Biosystems, Foster City, CA, USA) and SYBR Green chemistry. PCR conditions were as follows: initial denaturation at 95°C for 2 min, followed by 40 cycles of 95°C (30 s), 53°C (30 s), and 72°C (60 s) and a final extension phase at 72°C for 30 s followed by the generation of a dissociation curve to verify amplification specificity. Reactions contained 2.5 μL template DNA, 5 μL 2x Fast SYBR Green Master Mix (Applied Biosystems, Foster City, CA, USA), 0.3 μl forward and reverse primers (0.3 mM final concentration) and 1.9 μL nuclease-free H_2_O in a total volume of 10 μL. PCR efficiencies (E) were calculated as E = (10^−1/slope^ − 1) × 100.

To evaluate a potential PCR-bias for individual strains, DNA was extracted from 13 pure cultures isolated previously from the TNT-contaminated soil, including *Achromobacter* sp. zw108, *Arthrobacter* sp. zw99, *Bacillus licheniformis* zw124, *Chryseobacterium* sp. zw156, *Herbaspirillum* sp. zw98, *Nocardia* sp. zw144, *Paenibacillus* sp. zw125, *Pseudomonas* sp. zw136, *Rhizobium* sp. zw143, *Rhodococcus* sp. zw143, *Sphingobium* sp. zw115 and *Variovorax* sp. zw103. DNA was extracted from cultures using the Qiagen Blood and Tissue Kit according to the manufacturer's instructions (Qiagen, Venlo, Netherlands).

To compare the 454-sequence data with qPCR, the same soil samples were amplified using Bacteria domain and group specific qPCR primers (Supplementary Table 1). The log copy number per ng DNA and relative abundances were calculated using standard curves of plasmids. These plasmid-standards were prepared from *Escherichia coli, Okibacterium fritillariae* (phylum Actinobacteria), *Chryseobacterium vrystaatense* (phylum Bacteroidetes), *Bacillus mycoides* (phylum Firmicutes), *Novosphingobium barchaimii* (class Alphaproteobacteria), and *Burkholderia sediminicola* (class Betaproteobacteria). In brief, 16S rRNA gene amplicons of these strains were obtained by PCR amplification with the selected primers and cloned in the pGEM-T Vector (Promega Benelux, Leiden, the Netherlands). The vectors were incorporated in competent *E. coli* JM109 cells (Promega Benelux, Leiden, the Netherlands). Plasmids were isolated from successfully transformed cells using the UltraClean Standard Mini Plasmid Prep Kit (MO BIO Laboratories, Carlsbad, California, USA) and sent for Sanger sequencing (Macrogen Europe, Amsterdam, The Netherlands) to verify insert sequence. DNA concentrations of the plasmid suspensions were determined using Quant-iT PicoGreen (Life Technologies, Carlsbad, California, USA). Ten-fold serial dilutions of the plasmid suspensions (ranging from 10^1^ to 10^8^ copies per μl) were made in triplicate for all taxa including two no-template controls. PCR conditions used were: initial denaturation at 95°C for 15 min, 40 cycles of 94°C for 15 s, 30 s at the annealing temperature, 72°C for 30 s. Annealing temperatures were experimentally optimized to maximize the specificity of amplification (Supplementary Table 1). Each reaction was set up in triplicate. Reactions contained 2 μL DNA template, 5 μL of 2 x QuantiTect SYBR Green PCR Master Mix (Qiagen, Venlo, Netherlands), 0.2–0.8 μL of each primer [100–400 nM; depending on taxon (Supplementary Table 1)], and nuclease-free H_2_O in a total volume of 10 μL. Amplification was linear (*R*^2^ > 0.99) over six orders of dynamic range from 10^3^ to 10^8^ copies per μL, and efficiencies reached between 81 and 93.8% (Supplementary Figure 1). Melting curves were evaluated to confirm that the detected fluorescence originated from specific products and not from primer dimers or other amplification artifacts.

### *In silico* primer evaluation

To evaluate the primer-to-target mismatches *in silico*, primers were tested with PrimerProspector 1.0.1 (Walters et al., 2011) against the SILVA SSU ref NR database version 123 containing 597,607 16S/18S sequences (https://www.arb-silva.de/). All tests were performed as described by Walters et al. (2011) using standard settings. Primer scores were calculated based on the following formula: weighted score = non-3′ mismatches × 0.40 + 3′ mismatches × 1.00 + non-3′ gaps × 1.00 + 3′ gaps × 3.00. An additional penalty score of 3.00 was assigned if the final 3′ base of a primer had a mismatch with its target sequence (Walters et al., 2011). The closer the PrimerProspector value is to 0, the better the primers perform theoretically.

### Accession numbers

The standard flowgram format (SFF) files were deposited in the European Nucleotide Archive under study accession PRJEB18468 and sample accession numbers ERS1463796–ERS1463819.

## Results

### Read output and sequence length distribution of 454 amplicon pyrosequencing data

The 454 amplicon sequencing run generated a total of 116,739 raw reads with average raw read lengths of 420 bp for the primers tested. After read processing and quality filtering in QIIME, a total of 71,821 high quality bacterial reads were retained (Supplementary Table 2). The primer pairs 68f/518r, 341f/785r and 799f/1193r yielded an average of 14,442 sequences, and the dataset amplified with 967f/1391r had a total of 28,498 reads. The difference in read number was not related to difference in primer pair performance, as the amplicons were sequenced together with those of another unrelated study. The primers were found to be specific for Bacteria as almost no Eukaryotic or Archaeal sequences were amplified. Though some primer pairs were more likely to amplify chloroplast and mitochondrial reads, with 5,910 chloroplast reads for 68f-518r being the highest, and only 1 for 799f/1193r.

From the high quality read sequence length distribution graph it can be observed that primers 68f/518r and 341f/785r produced additional peaks next to the peak of the expected size, compared to primers 799f/1193r and 967f/1391r (Supplementary Figure 2A). The shorter amplicons with 490 bp length produced by 341f/785 were almost exclusively assigned to Alphaproteobacteria as shown by a blast search of 1,000 randomly selected sequences (Supplementary Figure 2A). Similarly, the peak of small amplicons produced by primer pair 68f/518r (410 bp) was dominated by reads assigned to Alphaproteobacteria, while the 440 and 460 bp sequences represented a more diverse set of bacterial taxa (Supplementary Figures 2A, 3). Examination of sequence length distribution showed that 98% of the shortest reads (high peak at 410 bp) were assigned to Rhizobiales, of which 89% were assigned to Bradyrhizobia, and 49% uncultured *Bradyrhizobium* sequences.

### Parametric analyses of pyrosequencing data

**Figure 1:**
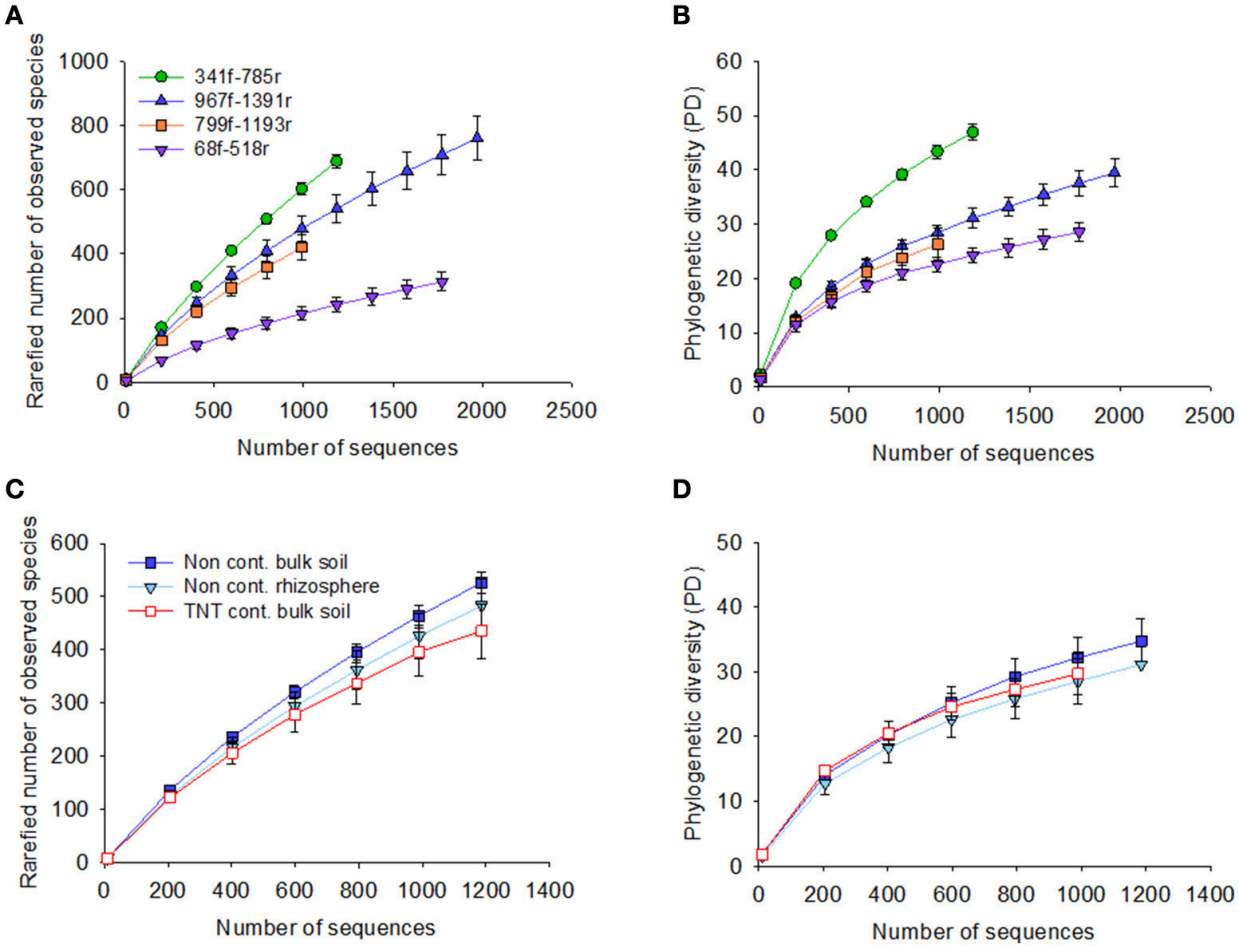
**Rarefaction curves for the four primer pairs used in this study, 68f/518r, 341f/785r, 799f/1193r, 967f/1391r**. Graphs show the rarefaction curves of 16S rRNA gene sequences based on pyrosequencing of the bulk soil and *Acer pseudoplatanus* rhizosphere soil samples collected from a military forest, Zwijndrecht, Belgium. The number of sequences were plotted against the rarefied number of observed species based on 97% sequence similarity cut-off value, and phylogenetic diversity for each of the primer pairs (**A**,**B**, respectively), and per soil type **(C,D)**.

**Figure 2:**
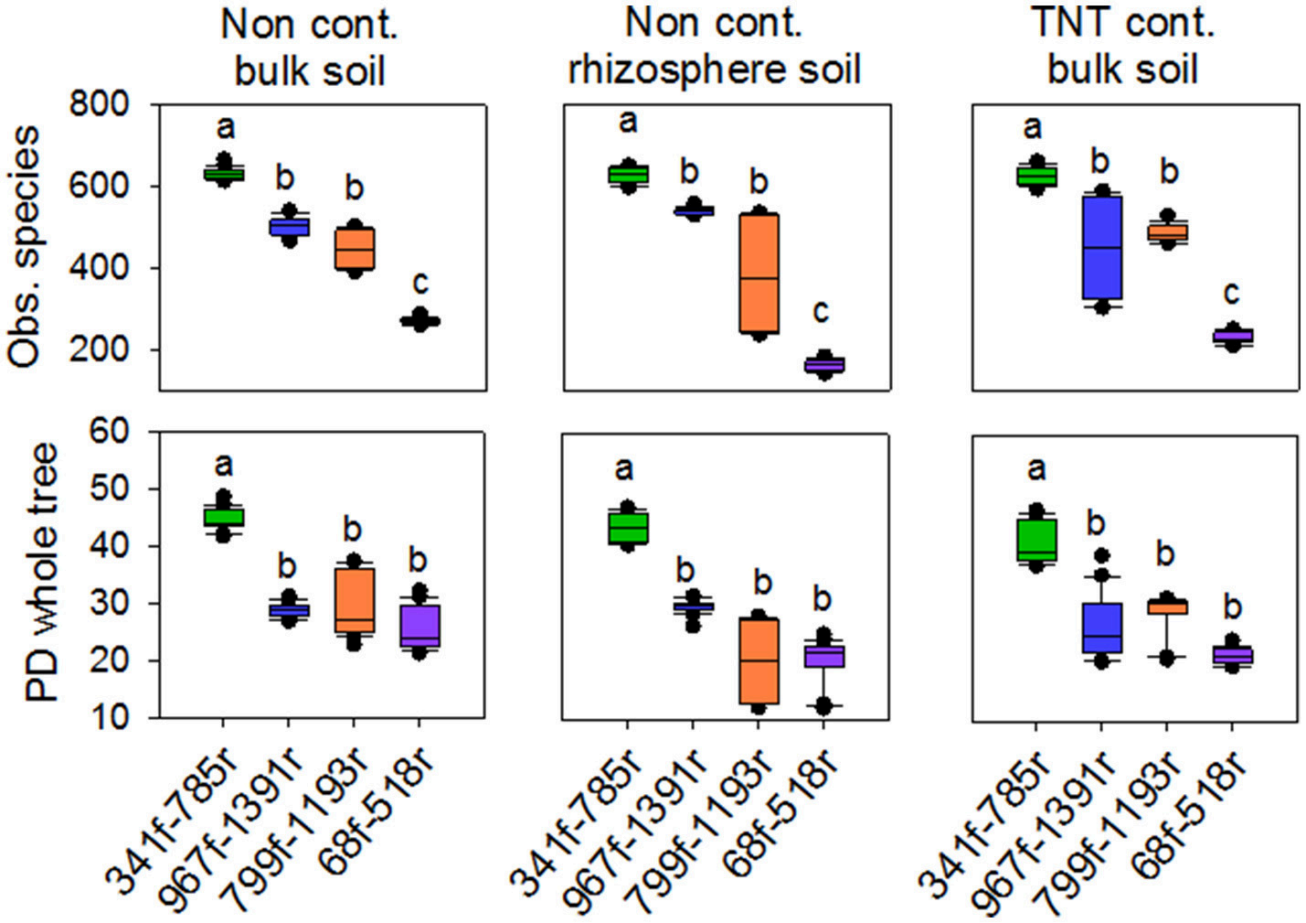
**Box plots of observed species and phylogenetic diversity for the four primer pairs used in this study, 68f/518r, 341f/785r, 799f/1193r, 967f/1391r**. Average number of observed species was based on 97% sequence similarity cut-off value. Averages were calculated based on a rarefied depth of 1,000 sequences per sample, across the replicates for each primer pair. Different letters denote significant differences (Kruskal Wallis, *p* < 0.05).

The 71,821 bacterial sequences that clustered at 97% identity threshold generated a total of 8,169 OTUs using the closed reference OTU picking strategy. Rarefaction curves for the total number of observed OTUs and phylogenetic diversity demonstrated that primer pair 341f/785r generated the steepest slope and highest diversity, followed by 967f/1391r, 799f/1193r, and 68f/518r (Figures 1A,B). The observed number of OTUs and Faiths PD index was significantly higher for 341f/785r compared to the other primer pairs (Kruskal–Wallis, *p* < 0.05) based on calculations with normalized reads (Figure 2) and non-normalized reads (Supplementary Figure 3). In detail, primer pair 341f/785r detected on average 599 ± 4.8 OTUs and the phylogenetic richness was 43 ± 0.3. While there was no significant difference in the number of observed species detected by primer pair 967r/1391r (493 ± 8.4) and 799f/1193r (401 ± 10), primer pair 68f/518r detected a significantly lower number (214 ± 5; Figure 2; Kruskal–Wallis, *p* < 0.05). The same trend was observed for the Chao richness, with the statistically highest values being for 341f/785r (1781 ± 183), followed by 967f/1391r (1306 ± 334), 799f/1193r (1112 ± 298), and 68f/518r (557 ± 103; Kruskal–Wallis, *p* < 0.05).

The rarefaction curves for the Shannon diversity and Inverse Simpson index revealed the same trends as observed for OTU richness and PD diversity (Supplementary Figures 4, 5). The Shannon diversity index value showed that primer pair 341f/785r had the highest diversity based on the most abundant bacteria, ranging from 8.4 to 8.9, while 68f/518r had the lowest, ranging from 3.2 to 5.4, and these differences were significant between the primer pairs (*p* < 0.05). Primer pairs 799f/1193r and 967f/1391r performed second best with similar Shannon indices, average of 7.76 ± 0.06. The inverse Simpson index showed the same trend as observed for the Shannon index.

Besides the alpha-diversity differences between the primer pairs, the data also revealed differences between the soil samples tested in the observed number of OTUs, Faith's PD, and diversity (Figures 1C,D). The non-contaminated bulk soil samples were more diverse than the TNT-contaminated bulk soil in terms of Shannon diversity and Inverse Simpson, as demonstrated by all primer pairs (Supplementary Table 3). Further, there were also primer pair dependent differences in taxon richness. Primer pair 341f/785r detected a significant lower taxon richness in the TNT-contaminated bulk soil samples (565 ± 12) compared to the non-contaminated bulk soil (622 ± 5) and rhizosphere samples (641 ± 14), while the other primer pairs detected only a decreasing trend (Supplementary Table 3). On the other hand, primer pair 967f/1391r detected a significantly higher number of OTUs in the rhizosphere (456 ± 4) compared to the bulk soil (427 ± 17), while no statistical difference was observed for primer pairs 341f/785r and 799f/1193r, and a significant decrease with 68f/518r.

### Phylum-level taxonomic distribution

**Figure 3:**
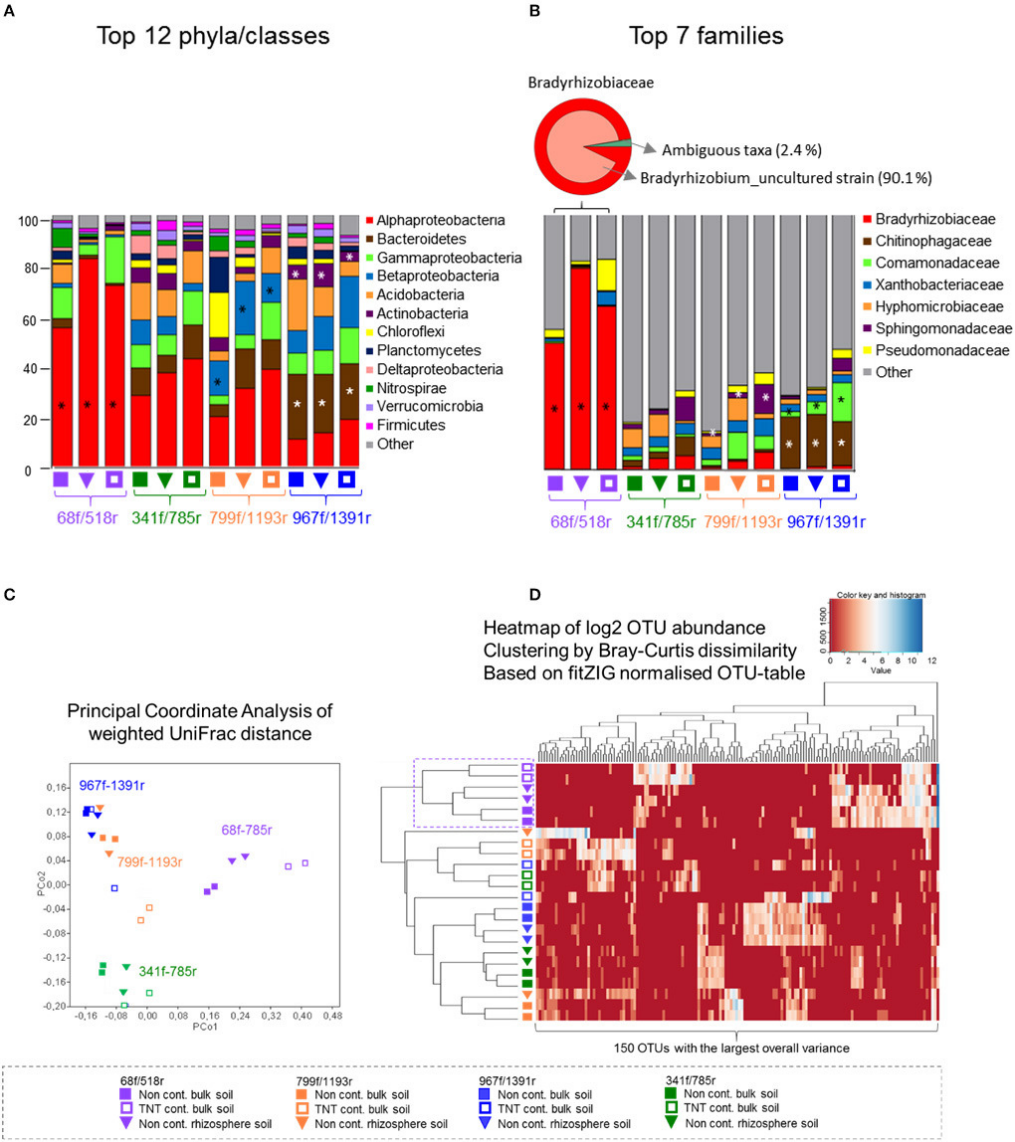
**Abundance distribution of the most dominant phyla, Proteobacteria classes, and families, principal coordinate analyses, and heatmap of the top 150 OTUs**. The relative abundance bar charts show the 12 most dominant phyla and Proteobacteria classes **(A)** and seven most dominant families **(B)** per primer pair and soil type, based on pyrosequencing data of bulk soil and *Acer pseudoplatanus* rhizosphere soil samples, Zwijndrecht, Belgium. Significant different lineages between the primers are indicated with a star (Kruskal–Wallis *p* < 0.05). **(C)** PcoA was calculated based on the CSS-normalized data and weigthed UniFrac distance matrix in QIIME. **(D)** The heatmap was generated based on the fitZIG normalized OTU-table (Paulson et al., 2013) and shows the 150 OTUs with largest overall variance clustered by Bray-Curtis dissimilarity.

All four primer pairs detected a different number of phyla and class level taxa. A total of 19 phyla were detected by 68f/518r, 27 by 341f/785r, 25 by 799f/1191r, and 28 by 967f/1391r (Figure 3A, Supplementary Table 4). The twelve most abundant phyla/classes that were detected by all primer pairs included Alphaproteobacteria, Bacteroidetes, Gammaproteobacteria, Betaproteobacteria, Acidobacteria, Actinobacteria, Chloroflexi, Planctomycetes, Deltaproteobacteria, Nitrospirae, Verrucomicrobia, and Firmicutes (Figure 3A). Some of the more abundant phyla not detected by primer pair 68f/518r in comparison to the other primer pairs were Chlamydiae, Chlorobi, and Gemmatimonadetes (Supplementary Table 4). Examples of primer pair differences in many lower abundance groups can be observed in Supplementary Table 4.

The relative abundances of the major phyla were also significantly different (Figure 3A). Primer pair 68f/518r was significantly biased toward Alphaproteobacteria (60–80% of the sequences, LDA-score > 4, LefSe) compared to the other primer pairs where the communities were more evenly distributed (Figure 3A). A statistically higher relative abundance of Bacteroidetes and Actinobacteria was detected by 967f/1391r compared to the other primer pairs (LDA-score > 4, LefSe *p* < 0.05), while Betaproteobacteria sequences were significantly more amplified by primer 799f/1193r (LDA-score > 4, LefSe *p* < 0.05). Interestingly, a comparison of the shared and single OTUs showed that only a small percentage of the OTUs were shared among all 4 primer pairs (0.5%; Supplementary Figure 6). Combining the data from 341f/785r, 799f/1193r, and 967f/1391r however covered over 90% of the OTUs.

Results of the weighted UniFrac distance PCoA plot demonstrated significant clustering of the samples according to the primer pair used rather than soil type (ANOSIM R = 0.82, *p* < 0.001; Figure 3C). Bray-Curtis cluster analyses of the 150 most dominant OTUs showed a separate grouping for 68f/518r, but the other primer pairs clustered in 1 group rather than 3 separate. Besides primer 68f/518r, the heatmap also showed that the TNT-contaminated soil samples clustered in a separate group from the non-contaminated bulk soil and rhizosphere samples with 75% Jackknife support, while the non-contaminated bulk soil and rhizosphere communities could not be significantly discriminated from each other (NPMANOVA, *p* < 0.26).

### Family, and genus-level PCR bias

The relative abundance of the 10 most abundant families detected differed between the primer pairs (Figure 3B). In total, 32 bacterial groups were distinct to at least one primer pair using the logarithmic (LDA) value of 4, and test-values of *p* < 0.05 (Supplementary Figure 7). Specifically, for primer pair 68f/518r, a bias was detected toward Bradyrhizobiaceae, *Bradyrhizobium*, and Rhizobiales (LDA score 5, LefSe at *p* < 0.05) within the Alphaproteobacteria, compared to the other primer sets. For primer pair 967f/1391r, Chitinophagaceae and Sphingobacteriales were detected more often, followed by *Terrimonas*, Burkholderiales, Actinobacteria, Micrococcales, and others (Supplementary Figure 7), while Flavobacteriaceae, and Sphingomonadaceae were detected more often with primer pair 799f/1193r. The fine-level bacterial groups that showed an LDA value higher than 4 with 341f/785r included Caulobacterales, Hyphomicrobiaceae, and Rhodospirillales.

Between soil samples, differences in the distribution of bacterial families and finer-level lineage were observed depending on the primer pairs used. Commonly for all primer pairs, 59 bacterial features (from phylum to species level) were found to differ between the uncontaminated bulk soil, TNT-contaminated bulk soil, and non-contaminated rhizosphere samples at an LDA of 2, *p* < 0.05. Supplementary Figure 8 shows the most significant features with LDA scores of 3.5 or higher, with Acidobacteriaceae/subgroup3, Acidobacteriales, Gammaproteobacteria, *Mizugakiibacter*, and *Rhodanobacter* enriched in the TNT-contaminated bulk soil, whereas *Nitrospira, Pedomicrobium*, and many uncultured bacteria belonging to other Acidobacterial groups were significantly enriched in the non-contaminated bulk soil, and Clostridia, Clostridiales, and Firmicutes were more enriched in the rhizosphere soils. We also noted the significantly enriched clades in the TNT-contaminated bulk soil for primer pair 341f/785r using Welch's *t*-test (*p* < 0.05) in STAMP (Supplementary Figure 9). This revealed an enrichment in the TNT-polluted soil of Xanthomonadaceae (Gammaproteobacteria), Bradyrhizobiaceae, Caulobacteracae, the Incertae Sedis group of the Rhizobiales (Alphaproteobacteria), and Chitinophagaceae (Bacteroidetes) amongst others, while several subgroups of the Acidobacteria, Deltaproteobacteria, and Betaproteobacteria were significantly depleted (Welch's *t*-test, *p* < 0.05; Supplementary Figure 9).

### Primer pair efficiency

The average PCR efficiencies (±standard error) were not statistically different between the primer pairs, and were calculated to be 96% (±1%) for 341f/785r, 105% (±2%) for 799f/1193r, 101% (±0.2%) for 68f/518r, and 99% (±0.6%) for 967f/1391r. Additionally, gel electrophoresis and dissociation curves were used to confirm the presence of amplicons of the expected size. Exponential amplification was obtained between 19 and 21 PCR cycles for all soil samples and primer pairs, and the dynamic range for the primer pairs spanned from 5 ng/μl to 50 pg/μl. The linear regression of log-concentration versus Cq values for the four primer pairs showed Spearman R^2^ values from 0.95 to 0.99. Similarly, no significant differences were found in amplification efficiency or sensitivity of the reference strains (Supplementary Figure 10). In some cases, primer pairs 967f/1391r and 341f/785r showed lower Cq values at exponential amplification (average of 17), compared to 21 for 68f-518r and 799f-1193r (average of 21), though not statistically significant.

### Reproducibility of bacterial OTU identification

Scatterplots and Spearman Rank coefficients (Supplementary Figure 11) showed that the duplicate biological soil samples were most consistently identified by primer pair 68f/518r (average r of 0.25), followed by 341f/785r (r of 0.21 for the rhizosphere samples and 0.25 to 0.29 for the bulk soils). Primer pair 799f/1193r showed reproducible identification of bacterial OTUs for the non-contaminated and TNT-contaminated bulk soil samples, but less for the rhizosphere samples (r 0.14), while primer pair 967f/1391r showed more variability between the biological replicates of TNT-contaminated samples (r 0.04).

### *In silico* primer evaluation

The *in silico* analyses of the primer pairs against the latest SILVA r123 database showed that the PrimerProspector score was best for primer 341f (0.07 ± 0.0005) and 785r (0.1 ± 0.0006), while a good overall score > 0.5 was obtained for 68f (0.83 ± 0.002) and 1391r (0.875 ± 0.002). The higher penalty score for 68f and 1391r was most likely caused by a higher occurrence of 3′ mismatches at the terminal base, namely 3.19 and 4.08% of the sequences, respectively. Primer 799f had also a higher percentage of sequences with 3′ end base mismatches (5.29%), and this was due to the sequences from Cyanobacteria (mismatch with 96% of sequences) and Verrucomicrobia (mismatch with 48% of sequences; Supplementary Table 5). For 341f, ~59% of Armatimonadetes sequences had a mismatch at the 3′ end terminus of the primer which can influence the amplification efficiency for this taxonomic group. For primer 68f, 40% of Chlorobi sequences showed a 3′ end mismatch. Overall, based on the average weighted PrimerProspector score and last base mismatches, the best primer pair would be 341f/785r, followed by 799f/1193r, 68f/518r, and lastly 967f/1391r.

**Figure 4:**
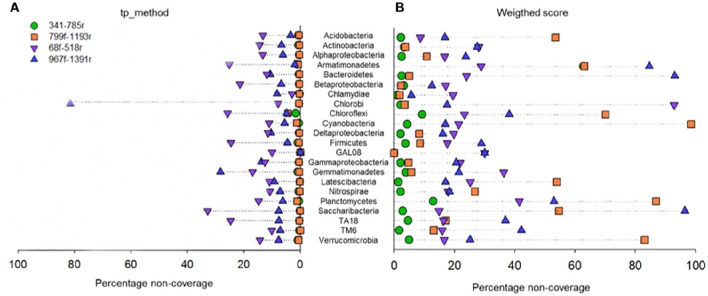
**Primer pair non-coverage percentage**. Calculated by the total primer mismatch method **(A)** or using the weighted score method **(B)** in PrimerProspector.

*In silico* taxonomic coverage of the primer pairs was predicted at the domain level (Table 1) and phylum level. The percentage non-coverage for the most abundant lineages (Figure 4) at the domain level showed that primer pair 341f/785r had the highest coverage for Bacteria representing 96.51% of the 159,615 bacterial sequences in the SILVA v123 database that were expected to be amplified (Table 1). The second best Bacterial domain coverage is expected from 799f/1193r (85.55%), followed by 68f/518r (78.59%), and 967f/1391r (66.54%). The coverage for the domains Archaea and Eukaryota was low (<1.37%) for most primer pairs.

At the phylum level, it can be observed that primer pair 341f/785r is theoretically able to amplify the most phyla, and this supports the findings from the experimental 454 pyrosequencing analyses (Figure 4). Depending on the scoring method used, total primer mismatch (tp_method) or average weighted score, different non-coverage rates were obtained (Figures 4A,B). In general, the tp_method in PrimerProspector has lower penalties, and tended to reflect better the sequencing results for the soil samples analyzed in this study. Using the tp_method, primer pairs 341f/785r and 799f/1193r showed the lowest non-coverage percentages (all <10%), while primer pairs 68f/518r and 967f/1391 had a non-coverage of 10% or higher for many phyla (Figure 4A). For example, primer pair 967f/1391r had a non-coverage percentage of 13.83% for Gammaproteobacteria, 28.3% for Gemmatimonadetes, and up to 81.25% for Chlorobi. Primer pair 68f/518r had a non-coverage percentage of 25.17% for Armatimonadetes, 21.37% for Betaproteobacteria, 25.79% for Chloroflexi, 24.53% for Firmicutes, 32.83% for Saccharibacteria, and 24.71% for TA18. Other groups that are not covered by 68f/518r include Chrysiogenetes, Sbxy-6786, Cver-2, Oscillatoria, and MD2896-b258, amongst others (Supplementary Table 4).

### qPCR analyses

**Figure 5:**
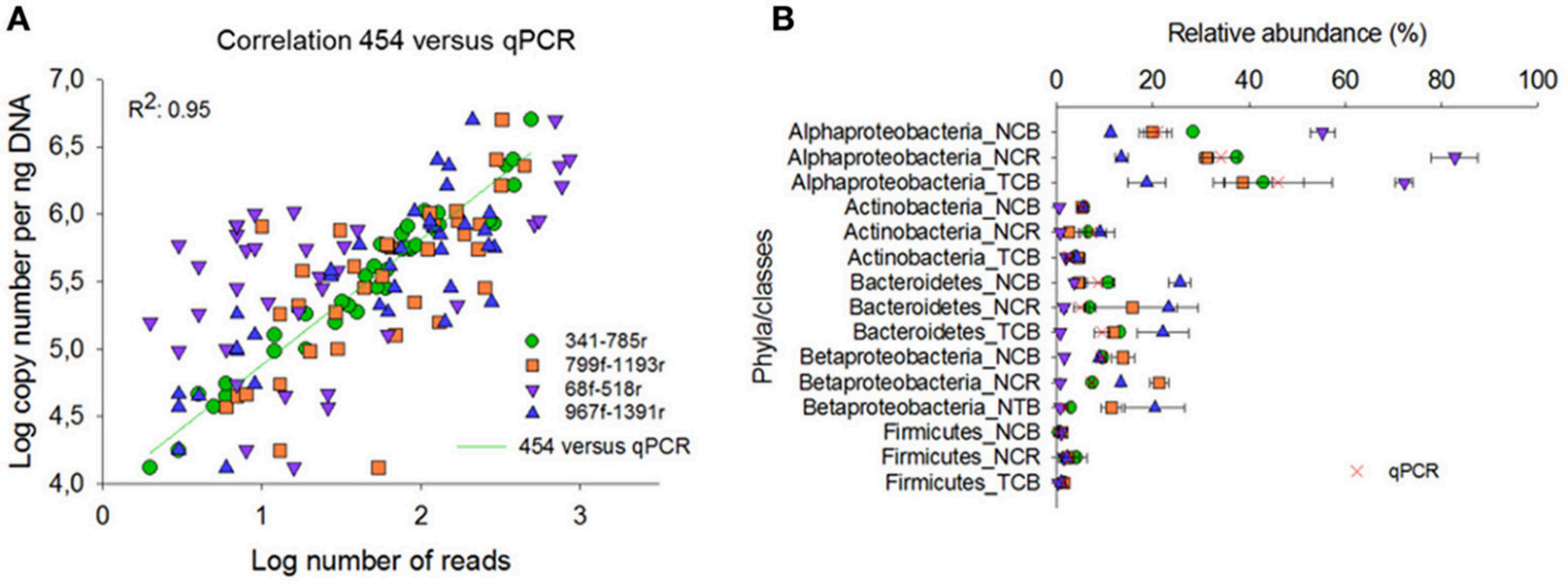
**Comparison of the relative abundance of different phyla and classes in the soil samples obtained by 454 pyrosequencing (A) and qPCR (B)**. NCB, non-contaminated bulk soil; NCR, non-contaminated rhizosphere soil; TCB, TNT-contaminated bulk soil.

Compared with the qPCR results, primer pair 341f/785r showed the best correlation Spearman rank coefficient (r 97.5), followed by 967f-1391r (72.8%), 799f-1193r (72.6%), and 68f-518r (38.9%; Figure 5A). The qPCR data were also expressed in relative percentage, by comparing phyla copy number to the copy number of total bacteria as estimated from the qPCR data of primer pairs EUB338f/EUB518r and Bact1369f/Prok1492r (Figure 5B). For all tested groups, the qPCR results supported best the relative abundances of bacterial phyla as estimated by primer pair 341f/785r using 454 sequencing technology (Figure 5B). The qPCR results confirmed that Alphaproteobacteria were overrepresented using primer pair 68f/518r, and underrepresented using 967f/1391r. Bacteroidetes were overrepresented in 967f/1391r using 454 compared to the qPCR results. Overall, for the taxonomic groups and soil samples tested here, primer pair 341f/785r is in best accordance with the qPCR results.

## Discussion

To address the need for bacteria-specific primer pairs in order to maximize overall coverage and phylum spectrum for specific applications, we evaluated the performance of four commonly used bacteria-specific primers for short-read sequencing using experimental and *in silico* approaches on environment-associated microbial communities. All primer sets were found to be highly specific for the domain Bacteria as almost no Archaea and Eukaryotic reads were amplified, and the theoretical coverage for the domains Archaea and Eukaryota was low (<1.37%). We demonstrated that primer pair 341f/785r was able to amplify from a heterogeneous pool of DNA and reveals the highest bacterial taxonomic coverage and diversity. Our study used the latest SILVA database v123 for *in silico* primer testing and taxonomic identification, and therefore adds value in the field of microbial community analyses as a similar comparative soil bacterial PCR-primer analyses has not been performed in recent years. We showed that primer pair 341f/785r performed well on 3 different soil types, including explosives contaminated soil samples, non-contaminated humus-rich forest soil samples and sycamore rhizosphere samples. This suggests a more wide-spread use of primer 341f/785r in bacterial community studies would enhance the comparability with other studies/environments in which 341f/785r has been commonly used, including mouse cecum samples, lettuce microbiome, bioremediation, and marine habitats. Though, while the study here provides a promising primer set, the long-term value will be in the methodology presented for evaluating primer sets as databases grow and improve, requiring temporal re-evaluation of the performance of primer pairs.

Primer pair 341f/785r had the highest average observed diversity per sample (609 ± 10 OTUs), significantly higher than the number of OTUs detected by other primer pairs (Figures 1, 2). Our results are therefore in good agreement with the values reported for a clay and loam soil in the UK (879 ± 21 to 2744 ± 124 OTUs), despite the much higher sequencing depth used in their study. The second best primer pair in our study, 967f/1391r, detected 479 ± 38 OTUs and this is in line with the results described by Beckers et al. (2016), with an average of 277 OTUs detected per sample for poplar rhizosphere, root, stem and leaf tissues. Primer set 799f/1193r revealed 438 ± 24 OTUs in the bulk soils compared to 383 ± 122 in the *A. pseudoplatanus* rhizosphere, suggesting that this chloroplast mismatch primer, which was effective in reducing chloroplast amplification for *A. pseudoplatanus*, comes at a trait-off in the number of OTUs detected and overall coverage and phylum spectrum. Previously, primer set 799f and 1193r was shown to effectively reduce chloroplast amplification from *A. thaliana* rhizosphere and root endophytic tissues ((Bulgarelli et al., 2012), (Schlaeppi et al., 2014)), seed endophytes (Truyens et al., 2015), and soybean, canola, and common bean leaves (Copeland et al., 2015). Interestingly, in our study, primer set 967f/1391r was also effective in reducing the co-amplification of chloroplast targets from *A. pseudoplatanus* rhizosphere samples (only 38 reads detected or <0.1%) while maintaining a higher overall coverage compared to 799f/1193r, suggesting it is a promising primer for bacterial community analyses of sycamore trees and potentially other plant species. Other commonly applied strategies to reduce amplification of chloroplast when amplifying microbial communities associated with an eukaryotic host is the use of peptide nucleic acid PCR clamps (Lundberg et al., 2013) or the post-sequence elimination of chloroplast reads given the upgrades of sequencing technology.

A difference noted between primer 341f/785r, 799f/1193r and 967f/1391r is the two amplicon sizes produced by 341f/785r (Supplementary Figure 2A) and a potential over-representation of Rhizobiales and Acidobacteria/Subgroup3 (Figure 3A). This effect was not noticed in studies using 341f/785r on marine microbial communities (Klindworth et al., 2013) or in other studies using the related primer pair 343f (Yergeau et al., 2014, 2015). We confirmed that the dual amplicon sizes were not due to sequencing artifacts, or platform effects (Supplementary Figure 12), but due to gaps in the 16S sequence at positions 454–465 and 476–485 (*E. coli* numbering) in many Alphaproteobacteria not present in the majority of other bacterial taxa (Supplementary Figure 2B). The shorter read length may favor the amplification of these subgroups by primer pair 341f/785r, however we cannot confirm this with the data presently available. Further analyses using shotgun metagenomics could evaluate the extent to which relative abundance differences of these taxa may be a problem. Previously, we used the bacteria/archaea primer pair 515f-806r to obtain amplicons from the same non-contaminated and TNT-contaminated bulk soil samples to identify the bacteria/archaea microbiome in the mycosphere of the fungus *Clitocybe nebularis* growing in the TNT-contaminated soil (Thijs, submitted), and we found that 515f/806r produced sequences of 1 length distribution (290 bp average read length), and the relative abundance of the most abundant taxa most closely resembled that generated with 341f/785r (Supplementary Figure 13). Primer pair 515f/806r also amplified archaeal reads as expected (94.1% coverage) representing a substantial number of reads in the dataset. An additional advantage of 341f/785r (V3–V4) compared to 515f/806r (V4) is the 1.5 times longer amplicon length and the coverage of two V-regions which can improve species level assignment (Baker et al., 2003; Vasileiadis et al., 2012; Lundberg et al., 2013). Rarefaction analyses also showed a higher phylogenetic diversity and higher number of observed species for 341f/785r compared to 515f/806r (Supplementary Figure 13), suggesting that for bacterial biodiversity studies, primer pair 341f/785r is more useful for bacterial community analyses by providing increased specificity for bacterial reads.

Principal coordinate analyses of the weighted UniFrac distance matrix revealed that the bulk soil and rhizosphere soil samples clustered per primer pair with high concordance (Figure 3C). Sample type specific differences were also observed from the dual dendrogram-heatmap with the rhizosphere and non-contaminated bulk soil clustering separate from the TNT-contaminated bulk soil samples for all primer pairs (Figure 3D). The relative abundance plots of the most abundant taxa confirmed the observations from the PCoA and heatmap, indicating significant differences in relative abundance across the phylum to genus level, suggesting primer-pair dependent amplification biases (Figure 3A). Notably, primer pair 68f/515r showed a large overrepresentation of Alphaproteobacteria and less coverage of all other taxa compared to the other primer pairs. The likely explanation for this observation is the poor predicted *in silico* coverage of primer pair 68f/518r for many taxa, leading to the preferential amplification of certain groups, while the other primer sets covered a larger number of phyla resulting in an increased number of reads that successfully clustered against the SILVA database.

Based on the *in silico* coverage and PrimerProspector score, primer pair 341f/785r scored best with 96.51% coverage of the domain Bacteria, and 0.07 ± 0.0005 and 0.1 ± 0.0006 average weighted scores for 341f and 785r, respectively (Table 2). For the *in silico* domain coverage prediction we used the weighted scoring in PrimerProspector and the latest SSU non-redundant reference SILVA database v123. Primer pair 341f/785r was developed against an earlier version of the SILVA database (release 108); here we show that the primer pair still attains a high taxonomic sensitivity against the updated SILVA database containing an additional 222,034 new sequences since 2013. The PrimerProspector score of 68f (0.71 ± 0.002) and 518r (0.08 ± 0.0005) was less optimal than 341f/785r, and this could be in part explained by the fact that 3% of the sequences had a mismatch in the last 3′ base of primer 68f which detrimentally affects amplification. For example, Chlorobi, an ubiquitous taxon in soil, had in 40% of the sequences a mismatch in the 3′ region of the 16S rRNA gene targeted by 68f, and this resulted in a complete absence of amplification. Next to Chlorobi, 68f/518r did not cover many other important groups in soil (Supplementary Table 4B) and this was reflected in the lowest Shannon diversity (ranging from 3.2 to 5.4) and number of OTUs (214 ± 5) for all soil samples. Previously, primer pair 68f/518r was used to amplify iron-oxidizing Zetaproteobacteria in soil but it has not been extensively validated as a primer for soil microbial community characterization (McAllister et al., 2011). Because primer 68f/515r cannot reliably assess Alphaproteobacteria and has a low coverage, we do not recommend this primer for soil microbiome analyses. For comparison, primer pair 799f/1193r (0.37 ± 0.001, 0.21 ± 0.0006) had a better PrimerProspector score, while 967f/1391 had the worst score (0.32 ± 0.0007 and 0.87 ± 0.002), though experimental results did not suggest that 967f/1391r performed the worst in terms of phylogenetic richness. For instance, *in silico* prediction suggested a high non-coverage for Bacteroidetes (Figure 4), but experimentally 967f/1391r detected significantly the highest relative abundance of Bacteroidetes, suggesting that care should be taken to interpret *in silico* analyses and experimental analyses are essential to support the findings.

**Table 2:** **Percentage of sequences with last 3′ base mismatch and PrimerProspector score**.

**Primer pair**	**Last base mismatch (%)**	**Average score ± SE**
341f	0.92	0.07 ± 0.0005
518r	0.41	0.08 ± 0.0005
785r	0.79	0.1 ± 0.0006
1193r	0.48	0.21 ± 0.0006
967f	0.34	0.32 ± 0.0007
799f	5.29	0.37 ± 0.001
68f	3.19	0.71 ± 0.002
1391r	4.08	0.87 ± 0.002

*The SILVA SSU non-redundant database release 123 was used for the analyses, which contains 597,607 bacterial sequences*.

The results obtained by 454 technology were also compared with qPCR analyses of the same soil samples to test whether bulk amplicon sequencing appropriately quantified the relative abundance of specific OTUs in the community (Figure 5). Primer pair 341f/785r approximated best the qPCR data using group-specific qPCR primer sets (Pfeiffer et al., 2014), followed by 967f/1391r and 799f/1193r. Indeed, the ratio between the bacterial 16S rRNA gene copies as determined by qPCR supported the relative amount of bacterial phyla as estimated from the 454 amplicon library (Figure 5), indicating that 341f/785r used for the 454-sequencing allowed a high rate of coverage and amplified with relatively little bias under the applied conditions. Additionally, further analyses, such as with mock communities, could evaluate the extent to which the primer pairs truly represent microbial community distributions. In addition, previous shotgun metagenome analyses have shown that 341f/785r reflects metagenome libraries (Klindworth et al., 2013).

In addition to presenting an approach to evaluate the performance of primer pairs to characterize soil bacterial microbiomes, our study also provides the first view of bacterial communities present at a TNT-contaminated forest soil (Figure 3A). The forest soil was dominated by Alphaproteobacteria (37.7%), Bacteroidetes (12.5%), Gammaproteobacteria (9.9%), Betaproteobacteria (9.6%), Acidobacteria (8.5%), and Actinobacteria (4.1%), an observation made using data generated from all four primer pairs. The TNT-polluted soil harbored a bacterial community that shifted toward a higher relative abundance of the Gammaproteobacteria (LDA > 4.5, *p* < 0.05) compared to the non-contaminated bulk soil (Supplementary Figure 8). Although different primer pairs indicated different dominant OTUs, a few bacterial groups were commonly identified as more abundant in the TNT-contaminated bulk soil including Acidobacteria/Subgroup1, Acidobacteriales, Mizugakiibacter, and Rhodanobacter. Primer pair 341f/785R detected an enrichment in the TNT-polluted soil of Xanthomonadaceae (Gammaproteobacteria), Bradyrhizobiaceae, Caulobacteracae, and alphaIcluster of the Rhizobiales (Alphaproteobacteria), and Chitinophagaceae (Bacteroidetes), suggesting these taxa are quite tolerant to high TNT-concentrations (>5%). A higher prevalence of Gamma- and Alpha-proteobacteria has been reported in a hydrocarbon contaminated soil (Yergeau et al., 2014), which suggest an r-strategy life-style for these taxa. With respect to TNT-contaminated soil, some of the groups detected in this study are in agreement with the results from clone libraries and DGGE-analyses of TNT-contaminated soil in the UK (Travis et al., 2008), and France (Eyers et al., 2006). Moreover, cultured strains of the Gamma- and Alpha-proteobacteria (Rhizobiales) have the ability to reduce trinitrotoluene to aminonitrotoluene (Labidi et al., 2001; Ramos et al., 2005; Chien et al., 2014). Importantly, we detected recently that the soil bacterium *Raoultella* (Enterobacteriaceae) has the ability to liberate nitrite from TNT for growth catalyzed by a protein of the Old Yellow Enzyme family, N-ethylmaleimide reductase (Thijs et al., 2014). The other taxa in this study which appear to have been enriched on TNT-contaminated soil have not been investigated in culture-conditions, and constitute promising candidates for further investigation.

## Conclusion

Using experimental and *in silico* approaches for bacterial community characterization, we found that among the four bacteria-specific primer pairs tested in this study, 341f/785r is the most promising set for bacterial community analyses because it showed the highest taxonomic coverage, diversity, reproducibility, PCR-amplification efficiency and best agreement with qPCR data on the tested soil samples. The primers used in this study are also applicable to be used on the Illumina platform and Ion Torrent. Knowledge on how various primer pairs compare and amplify taxa is of critical importance and must be considered as part of a robust experimental design. Together temporal re-evaluation of primer pairs against up-to-date databases is important in order to best represent our understanding of microbial taxonomy.

## Author contributions

Based on his experience in primer design and testing, MO contributed significantly in the design of the work and critical revision of the article. BB, STr, and VS were of great help for the practical design of the work including next generation sequencing library preparation, and data collection. STh designed the work, performed data collection and interpretation, and wrote the paper. JDV has great experience in sequencing data analyses and helped with the interpretation and critical revision of the article, and English language correction. JV and NW are authorities in the field of bioremediation research and both gave their approval and critical evaluation of the final version to be published.

### Conflict of interest statement

The authors declare that the research was conducted in the absence of any commercial or financial relationships that could be construed as a potential conflict of interest.
