# Supplementary material for: Comparative Evaluation of Four Bacteria-Specific Primer Pairs for 16S rRNA Gene Surveys
Source: Front Microbiol. 2017 Mar 28;8:494. doi: 10.3389/fmicb.2017.00494 (PMC5368227; doi:10.3389/fmicb.2017.00494)
Supplement: Supplementary file 1 [file Table1.PDF]

Supplementary Table 1: General and group-specific bacterial qPCR primers

Taxon	Primer	Sequence (5'→3')	Primer conc.	Annealing temp.	Reference
Bacteria	Eub338F	ACTCCTACGGGAGGCAGCAG	400 nM	50°C	Fierer *et al.* (2005)
	Eub518R	ATTACCGCGGCTGCTGG			
Bacteria	Bact1369F	CGGTGAATACGTTCYCGG	400 nM	50°C	Suzuki *et al.* (2000)
	Prok1492R	GGWTACCTTGTTACGACTT			
Actinobacteria	Acti1154F	GADACYGCCGGGGTYAACT	100 nM	55°C	Pfeiffer *et al.* (2014)
	Acti1339R	TCWGCGATTACTAGCGAC			
Bacteroidetes	Bdet107F	GCACGGGTGMGTAACRCGTAT	100 nM	55°C	Pfeiffer *et al.* (2014)
	Bdet309R	GTRTCTCAGTDCCARTGTGGG			
Firmicutes	Firm352F	CAGCAGTAGGGAATCTTC	100 nM	55°C	Pfeiffer *et al.* (2014)
	Firm525R	ACCTACGTATTACCGCGG			
Alphaproteobacteria	aProt528F	CGGTAATACGRAGGGRGYT	400 nM	55°C	Pfeiffer *et al.* (2014)
	aProt689R	CBAATATCTACGAATTYCACCT			
Betaproteobacteria	bProt972F	CGAARAACCTTACCYACC	300 nM	55°C	Pfeiffer *et al.* (2014)
	bProt1221	GTATGACGTGTGWAGCC			
