# Supplementary material for: Comparative Evaluation of Four Bacteria-Specific Primer Pairs for 16S rRNA Gene Surveys
Source: Front Microbiol. 2017 Mar 28;8:494. doi: 10.3389/fmicb.2017.00494 (PMC5368227; doi:10.3389/fmicb.2017.00494)
Supplement: Supplementary file 2 [file Table2.PDF]

Supplementary Table 2: Pyrosequencing raw reads and filtering. Table split by primer pair and summarising raw reads and quality filtered read statistics, processed in QIIME v1.9.1.

Raw reads and filtering				
**Primer pair**	**68f/518r**	**341f/785r**	**799f/1193r**	**967f/1391r**
**Number of samples**	**6**	**6**	**6**	**6**
**Raw data**				
Raw reads	27,361	24,792	22,742	41,844
Mean raw read length (bp)	427	423	402	431
**Quality filters (QF)**				
Read length outside bonds	4,272 (15.6 %)	2,357 (9.5 %)	3,128 (13.7 %)	3,594 (8.5 %)
Max homopolymer run > 6	62 (0.2 %)	461 (1.8 %)	47 (0.2 %)	306 (0.7 %)
Max primer mismatch > 4[a]	314 (1.1 %)	416 (1.7 %)	1,862 (8.1 %)	397 (0.9 %)
Mean quality score below 25	18 (0.06 %)	34 (0.1 %)	13 (0.05 %)	14 (0.03 %)
Total	4,666 (17.0 %)	3,268 (13.1 %)	5,050 (22.2 %)	4,311 (10.3 %)
**USEARCH_61 de novo chimera detection**				
Denovo chimeras[b]	2,615 (9.5 %)	2,785 (11.2 %)	2,967 (13.0 %)	8,973 (21.4 %)
**Post-filtering**				
Final reads	20,080 (73.4 %)	18,740 (75.6 %)	14,725 (64.7 %)	28,560 (68.3 %)
Archaea	0	5	4	0
Eukaryota	4	0	0	19
Chloroplast	5,91 (21.6 %)	3,986 (16.1 %)	1 (0.0 %)	38 (0.1 %)
Mitochondria	22	286	1	8
Final high quality bacteria reads	14,144 (51.7 %)	14,463 (58.3%)	14,719 (64.7%)	28,495 (68.1 %)
Trimmed read length (bp)	300	300	300	300

[a] The --max-primer-mismatch value was changed from the default value of 0 to 4 to account for the degenerate bases in the primer

[b] Usearch61_minh was set to 1 as recommended (Edgar et al. 2011).
