# Supplementary material for: Comparative Evaluation of Four Bacteria-Specific Primer Pairs for 16S rRNA Gene Surveys
Source: Front Microbiol. 2017 Mar 28;8:494. doi: 10.3389/fmicb.2017.00494 (PMC5368227; doi:10.3389/fmicb.2017.00494)
Supplement: Supplementary file 3 [file Table3.PDF]

Supplementary Table 3: Alpha diversity metrics per primer pair and soil type

Alpha-diversity indices per primer pair and soil type				
	68f/518r	341f/785r	799f/1193r	967f/1391r
**Observed species**				
Non cont. bulk soil	274 ± 4 a	622 ± 5 a	447 ± 37 a	427 ± 17 a
Non cont. rhizosphere soil	160 ± 4 b	641 ± 14 a	383 ± 122 a	456 ± 4 b
TNT cont. bulk soil	209 ± 9 a	565 ± 12 b	429 ± 11 a	343 ± 94 a
**Phylogenetic diversity**				
Non cont. bulk soil	26 ± 0.8 a	45 ± 0.4 a	29 ± 1.2 a	28 ± 0.3 a
Non cont. rhizosphere soil	20 ± 0.9 b	44 ± 0.5 a	20 ± 1.6 b	29 ± 0.3 b
TNT cont. bulk soil	22 ± 0.3 b	42 ± 0.8 b	29 ± 0.8 a	26 ± 1.2 a
**Shannon diversity**				
Non cont. bulk soil	5.4 ± 0.03 a	8.9 ± 0.01 a	7.5 ± 0.1 a	8.2 ± 0.03 a
Non cont. rhizosphere soil	3.2 ± 0.07 b	8.8 ± 0.02 b	7.3 ± 0.23 a	8.4 ± 0.02 a
TNT cont. bulk soil	4.7 ± 0.04 c	8.4 ± 0.02 c	7.8 ± 0.02 b	7.4 ± 0.23 b
**Inv. Simpson**				
Non cont. bulk soil	6.5 ± 0.3 a	344 ± 1 a	52 ± 7.3 a	107 ± 1.8 a
Non cont. rhizosphere soil	2.5 ± 0.09 b	292 ± 8.5 b	78 ± 27.3 a	129 ± 4.8 a
TNT cont. bulk soil	5.6 ± 0.4 c	154 ± 6.3 c	108 ± 0.39 b	109 ± 27.4 a

Diversity metrics showing observed species, phylogenetic diversity, Shannon diversity, and Inverse Simpson metric for the bulk soil and rhizosphere forest soil samples, Zwijndrecht, Belgium. Averages were calculated based on the rarefied OTU-table. Different letters denote significant differences (Kruskal Wallis, $p < 0.05$).
