# Supplementary material for: Comparative Evaluation of Four Bacteria-Specific Primer Pairs for 16S rRNA Gene Surveys
Source: Front Microbiol. 2017 Mar 28;8:494. doi: 10.3389/fmicb.2017.00494 (PMC5368227; doi:10.3389/fmicb.2017.00494)
Supplement: Supplementary file 4 [file Table4.PDF]

Supplementary Table 4: Percent composition per primer pair and sample for the higher abundant (> 1 % RA) and lower abundant lineages (< 1 % RA).

a

Phyla/classes (> 1 % RA)	68f/518r			341f/785r			799f/1193r			967f/1391r		
	Non cont. bulk soil	Non cont. Rhizosphere soil	TNT cont. bulk soil	Non cont. bulk soil	Non cont. Rhizosphere soil	TNT cont. bulk soil	Non cont. bulk soil	Non cont. Rhizosphere soil	TNT cont. bulk soil	Non cont. bulk soil	Non cont. Rhizosphere soil	TNT cont. bulk soil
Acidobacteria	8.08	1.21	1.52	15.35	9.90	13.18	3.84	2.98	10.71	20.05	12.73	5.91
Actinobacteria	0.51	0.56	2.02	5.86	6.77	4.29	5.30	2.42	4.34	5.51	9.24	4.49
Alphaproteobacteria	53.89	81.82	72.93	28.13	38.74	42.58	20.35	31.06	38.84	11.52	13.64	18.03
Armatimonadetes	0.00	0.05	0.15	0.10	0.10	0.05	0.00	0.15	0.40	0.10	0.15	0.71
Bacteroidetes	3.69	1.67	0.81	11.01	6.77	13.13	4.70	15.96	11.77	26.01	22.27	23.08
Betaproteobacteria	1.77	0.76	0.66	9.75	7.68	3.18	13.99	21.01	11.26	8.38	13.43	21.11
Chlamydiae	0.00	0.00	0.00	0.00	0.00	0.05	0.10	2.07	0.30	0.66	0.96	4.95
Chlorobi	0.00	0.00	0.00	0.10	0.05	0.00	0.61	0.71	0.10	0.10	0.05	0.00
Chloroflexi	1.77	0.30	0.00	3.18	3.18	0.45	17.93	3.79	0.86	2.78	2.42	0.25
Cyanobacteria	0.25	3.94	0.20	0.05	0.71	0.35	0.05	0.05	0.00	0.10	0.00	0.30
Deltaproteobacteria	1.31	0.15	0.20	7.37	5.56	1.36	2.37	2.88	0.51	3.79	2.83	1.11
Firmicutes	0.76	1.82	0.35	0.45	4.04	1.57	1.31	2.42	1.67	0.61	1.72	0.71
Gammaproteobacteri	12.12	4.44	18.18	9.55	7.53	14.09	3.69	5.86	14.80	8.08	9.55	13.48
Gemmatimonadetes	0.00	0.00	0.00	0.51	0.05	0.00	0.30	0.00	0.15	0.86	0.66	0.56
Latescibacteria	0.05	0.00	0.00	0.56	0.05	0.00	0.00	0.00	0.00	0.51	0.30	0.00
Nitrospirae	8.38	0.71	0.00	2.07	1.72	0.30	5.91	2.88	0.00	1.46	2.47	0.00
Planctomycetes	3.74	0.20	0.05	2.02	2.27	1.92	13.89	1.31	0.30	4.85	3.08	1.77
Saccharibacteria	0.45	0.56	1.82	0.45	0.51	0.66	1.26	2.02	1.87	0.51	0.40	1.06
TA18	0.20	0.15	0.05	0.10	0.05	0.10	0.30	0.00	0.30	0.25	0.35	0.00
TM6	0.96	0.61	0.66	0.05	0.30	0.05	0.10	0.20	0.30	0.05	0.10	0.05
Verrucomicrobia	1.92	0.96	0.30	2.68	3.84	1.72	1.62	1.97	1.11	3.43	3.33	1.87

b

Phyla/classes (< 1 % RA)	68f/518r			341f/785r			799f/1193r			967f/1391r		
	Non cont. bulk soil	Non cont. Rhizosphere soil	TNT cont. bulk soil	Non cont. bulk soil	Non cont. Rhizosphere soil	TNT cont. bulk soil	Non cont. bulk soil	Non cont. Rhizosphere soil	TNT cont. bulk soil	Non cont. bulk soil	Non cont. Rhizosphere soil	TNT cont. bulk soil
Aerophobetes	0.00	0.00	0.00	0.00	0.05	0.00	0.00	0.00	0.00	0.05	0.00	0.00
Aminicenantes	0.05	0.00	0.00	0.00	0.00	0.00	0.00	0.00	0.00	0.00	0.00	0.00
ARKDMS-49	0.00	0.00	0.00	0.00	0.00	0.00	0.00	0.00	0.00	0.05	0.10	0.00
ARKICE-90	0.00	0.00	0.05	0.00	0.00	0.00	0.15	0.10	0.05	0.00	0.05	0.15
Candidate division OF	0.00	0.00	0.00	0.00	0.00	0.00	0.00	0.00	0.00	0.05	0.00	0.00
Elev-16S-509	0.00	0.00	0.00	0.00	0.00	0.00	0.00	0.00	0.00	0.00	0.05	0.00
Elusimicrobia	0.00	0.00	0.00	0.05	0.00	0.00	0.05	0.05	0.05	0.00	0.00	0.05
GAL08	0.00	0.00	0.00	0.00	0.00	0.00	1.82	0.00	0.00	0.00	0.00	0.00
Hydrogenedentes	0.00	0.00	0.00	0.00	0.00	0.00	0.00	0.00	0.00	0.05	0.00	0.00
JL-ETNP-Z39	0.00	0.00	0.00	0.05	0.00	0.00	0.00	0.00	0.00	0.05	0.00	0.00
Lentisphaerae	0.00	0.00	0.00	0.00	0.00	0.00	0.10	0.00	0.00	0.15	0.00	0.00
Microgenomates	0.10	0.05	0.00	0.00	0.00	0.00	0.00	0.00	0.00	0.00	0.00	0.00
Parcubacteria	0.00	0.00	0.00	0.10	0.00	0.05	0.20	0.00	0.00	0.00	0.05	0.00
Spirochaetae	0.00	0.00	0.00	0.00	0.05	0.00	0.00	0.10	0.00	0.00	0.00	0.00
TA06	0.00	0.00	0.00	0.30	0.10	0.00	0.00	0.00	0.00	0.00	0.05	0.00
Tenericutes	0.00	0.00	0.05	0.00	0.00	0.25	0.00	0.00	0.00	0.00	0.00	0.00
WCHB1-60	0.00	0.00	0.00	0.10	0.00	0.15	0.05	0.00	0.00	0.00	0.00	0.00
WD272	0.00	0.00	0.00	0.00	0.00	0.25	0.00	0.00	0.00	0.00	0.00	0.00

Averages were calculated based on a rarefied depth of 1,000 sequences per sample, across the replicates for each primer pair. Lineages that are not detected by some of the primer pairs are indicated in red.
