# Supplementary material for: Comparative Evaluation of Four Bacteria-Specific Primer Pairs for 16S rRNA Gene Surveys
Source: Front Microbiol. 2017 Mar 28;8:494. doi: 10.3389/fmicb.2017.00494 (PMC5368227; doi:10.3389/fmicb.2017.00494)
Supplement: Supplementary file 5 [file Table5.PDF]

Supplementary Table 5: Percentage of sequences with last 3’ base mismatch.

		last base mismatch (% of sequences)							
Phylum/class	Total number of sequences	68f	518r	341f	785r	799f	1193r	967f	1391r
Acidobacteria	13177	2	0	0	0	1	0	0	2
Actinobacteria	51160	14	1	1	1	1	0	0	4
Alphaproteobacteria	52738	3	0	0	1	9	1	0	2
Armatimonadetes	878	2	0	59	0	3	0	0	19
Bacteroidetes	49175	2	0	1	0	1	0	0	10
Betaproteobacteria	36239	1	0	0	1	1	0	0	4
Chlamydiae	375	2	1	0	0	0	0	0	0
Chlorobi	963	40	0	1	0	1	1	0	8
Chloroflexi	8809	2	0	1	1	3	0	0	17
Cyanobacteria	11379	6	1	1	1	96	1	1	2
Deltaproteobacteria	15232	3	0	0	0	1	1	0	3
Firmicutes	138523	2	1	1	1	2	0	0	2
Gammaproteobacteria	90471	2	0	0	1	1	0	0	5
Gemmatimonadetes	2029	2	0	0	1	2	1	0	3
Latescibacteria	476	2	0	0	0	1	1	0	1
Nitrospirae	1619	4	0	0	0	1	0	0	7
Planctomycetes	8037	3	0	1	0	4	1	0	4
Saccharibacteria	1203	1	0	2	0	1	0	0	8
TA18	263	3	0	2	2	3	0	0	8
TM6	534	8	0	0	1	1	1	0	1
Verrucomicrobia	3821	4	0	0	3	48	0	1	3
