# Supplementary material for: Comparative Evaluation of Four Bacteria-Specific Primer Pairs for 16S rRNA Gene Surveys
Source: Front Microbiol. 2017 Mar 28;8:494. doi: 10.3389/fmicb.2017.00494 (PMC5368227; doi:10.3389/fmicb.2017.00494)
Supplement: Supplementary file 6 [file Image1.PDF]

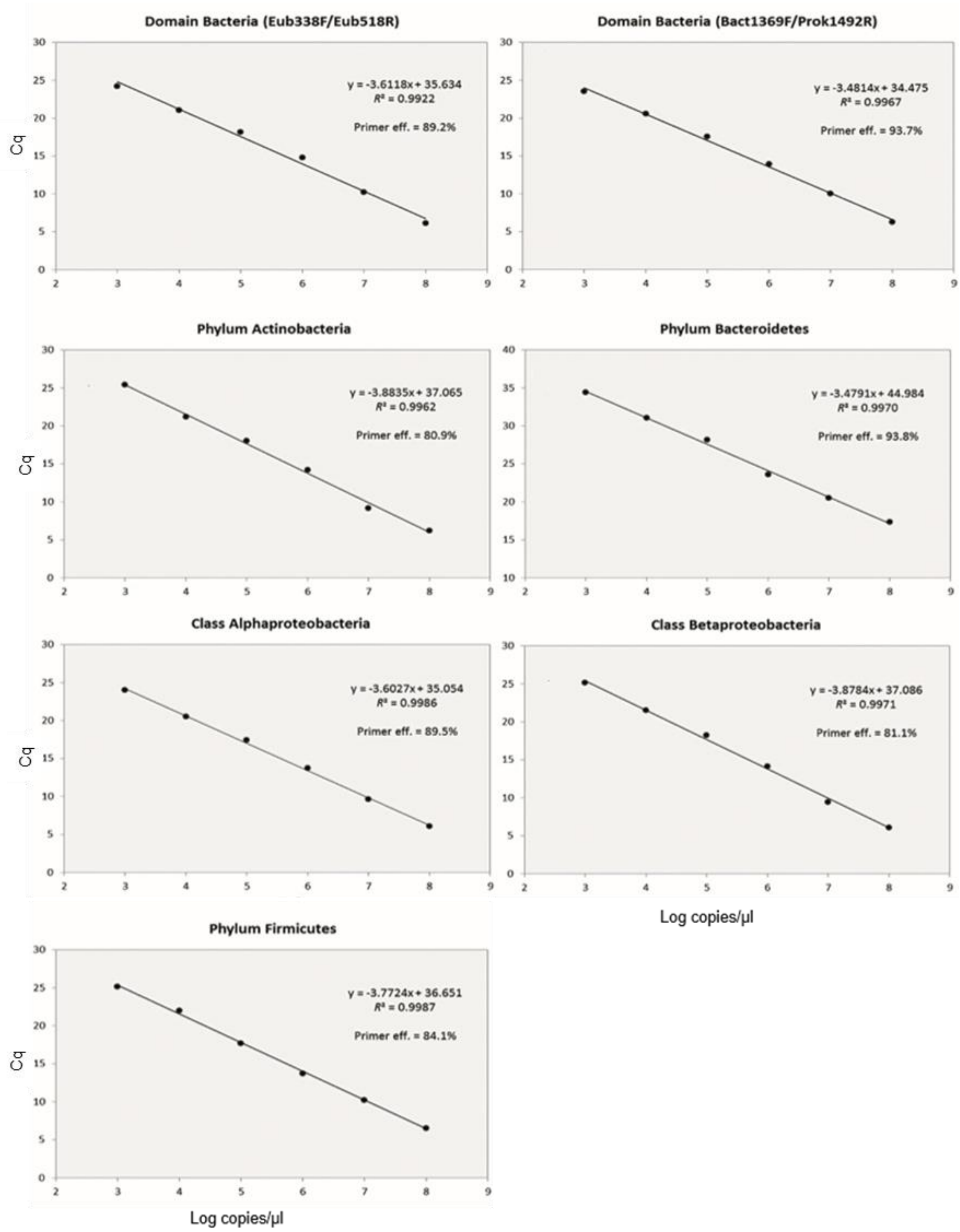

Supplementary Figure 1: qPCR primer efficiency of Bacterial domain and group-specific primers. Standard curves were made with plasmid DNA-standards cloned from *Escherichia coli* (Domain Bacteria), *Okibacterium fritillariae* (phylum Actinobacteria), *Chryseobacterium vrystaatense* (phylum Bacteroidetes), *Bacillus mycoides* (phylum Firmicutes), *Novosphingobium barchaimii* (class Alphaproteobacteria), and *Burkholderia sediminicola* (class Betaproteobacteria). PCR amplification of all primers was linear (R^2 > 0.99) over six orders of dynamic range from 10^3 to 10^8 copies per µL, and efficiencies were between 81 and 93.8 %.
