# Supplementary material for: Comparative Evaluation of Four Bacteria-Specific Primer Pairs for 16S rRNA Gene Surveys
Source: Front Microbiol. 2017 Mar 28;8:494. doi: 10.3389/fmicb.2017.00494 (PMC5368227; doi:10.3389/fmicb.2017.00494)
Supplement: Supplementary file 7 [file Image2.PDF]

a

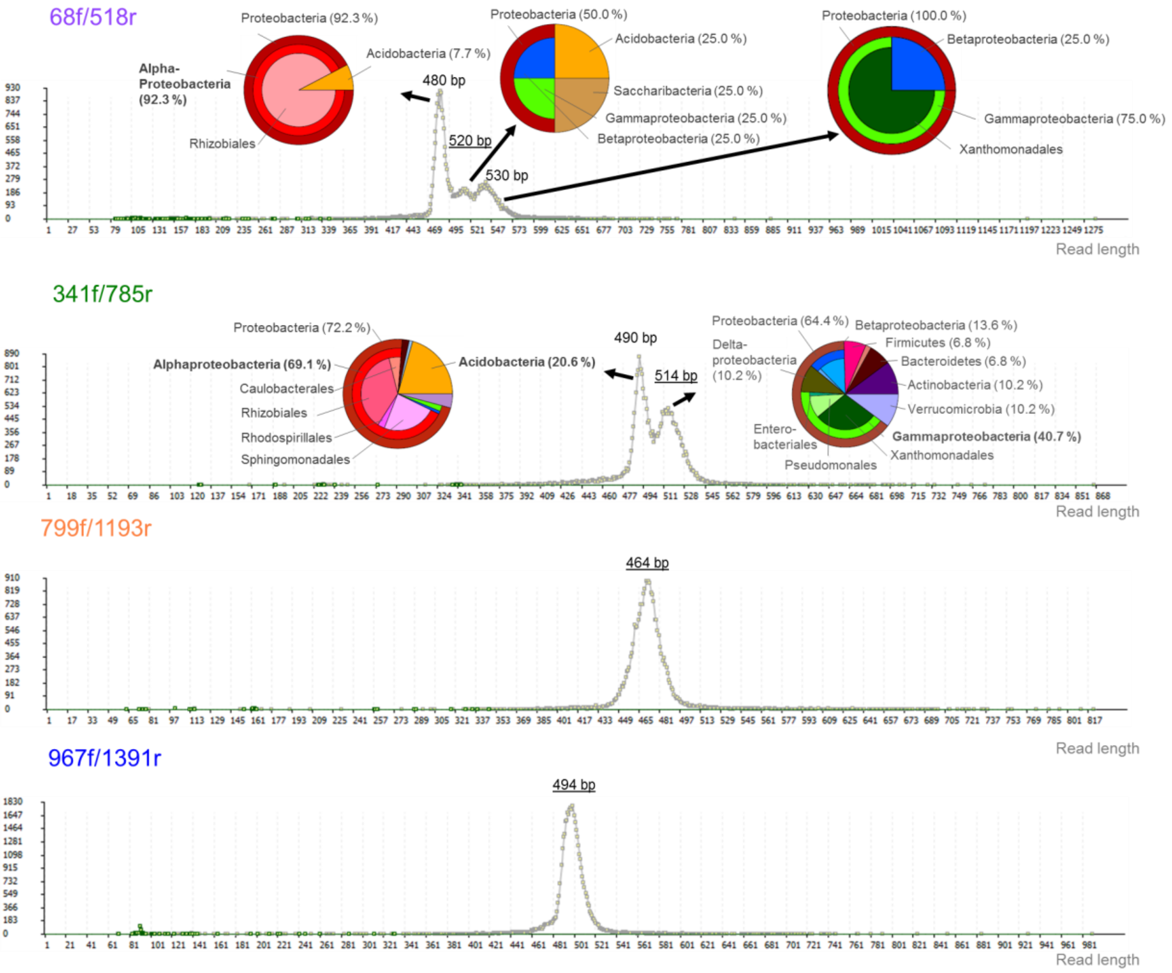

b

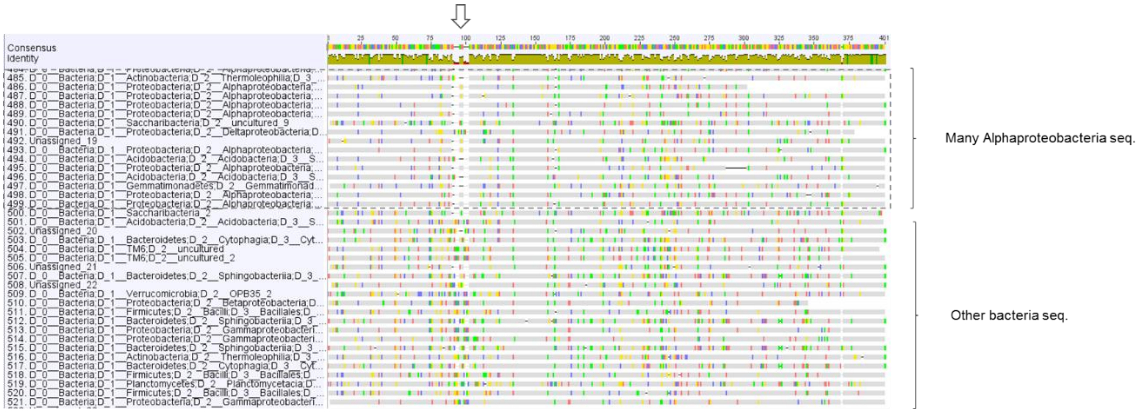

Supplementary Figure 2: Raw sequence length distribution per primer pair and multiple sequence alignment. Graphs show the raw 16S rRNA gene sequences for each primer pair as generated by pyrosequencing of the bulk soil and *Acer pseudoplatanus* rhizosphere soil samples collected from a military forest, Zwijndrecht, Belgium (**a**). Pie charts show the taxonomic distribution of 1,000 randomly picked quality filtered 16S sequences blasted against the SILVA v123 database. The picture at the bottom shows a snapshot of the multiple sequence alignment of 1,000 randomly picked 16S sequences from the 490 bp peak and 514 bp peak of 341f/785r (**b**). The arrow indicates the gaps in the 16S sequence at positions 454-465 and 476-485 (*E. coli* numbering) in Alphaproteobacteria whereas this was not detected in other bacterial taxa.
