# Supplementary material for: Comparative Evaluation of Four Bacteria-Specific Primer Pairs for 16S rRNA Gene Surveys
Source: Front Microbiol. 2017 Mar 28;8:494. doi: 10.3389/fmicb.2017.00494 (PMC5368227; doi:10.3389/fmicb.2017.00494)
Supplement: Supplementary file 8 [file Image3.PDF]

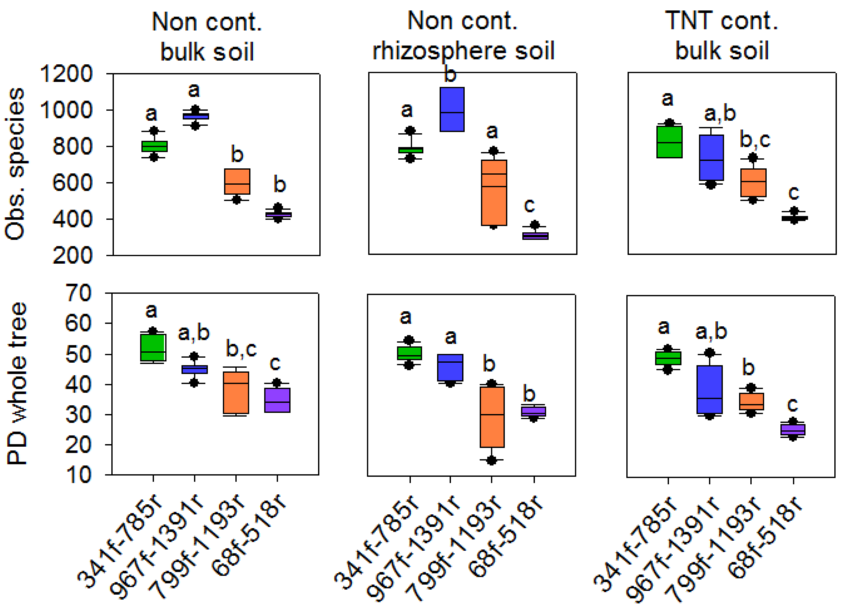

Supplementary Figure 3: **Box plots of observed species and phylogenetic diversity estimates for the four primer pairs used in this study, 68f/518r, 341f/785r, 799f/1193r, 967f/1391r**. Averages were calculated based on the non-normalized OTU-table. Different letters denote significant differences (Kruskal Wallis, $p < 0.05$).
