# Supplementary material for: Comparative Evaluation of Four Bacteria-Specific Primer Pairs for 16S rRNA Gene Surveys
Source: Front Microbiol. 2017 Mar 28;8:494. doi: 10.3389/fmicb.2017.00494 (PMC5368227; doi:10.3389/fmicb.2017.00494)
Supplement: Supplementary file 9 [file Image4.PDF]

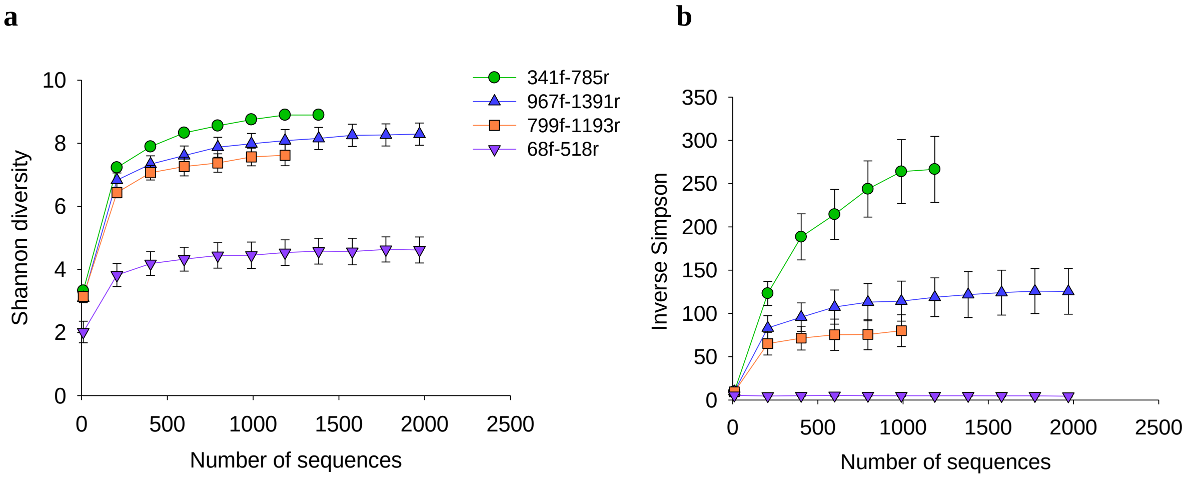

Supplementary Figure 4: **Rarefaction curves for the four primer pairs tested in this study, 68f/518r, 341f/785r, 799f/1193r, and 967f/1391r**. Graphs show the rarefied Shannon diversity (**a**) and Inverse Simpson diversity (**b**) for each primer pair based on pyrosequencing of the bulk soil and *Acer pseudoplatanus* rhizosphere soil samples collected from a military forest, Zwijndrecht, Belgium.
