# Supplementary material for: Comparative Evaluation of Four Bacteria-Specific Primer Pairs for 16S rRNA Gene Surveys
Source: Front Microbiol. 2017 Mar 28;8:494. doi: 10.3389/fmicb.2017.00494 (PMC5368227; doi:10.3389/fmicb.2017.00494)
Supplement: Supplementary file 10 [file Image5.PDF]

a. Non-normalized OTU-table: Shannon diversity and inverse Simpson

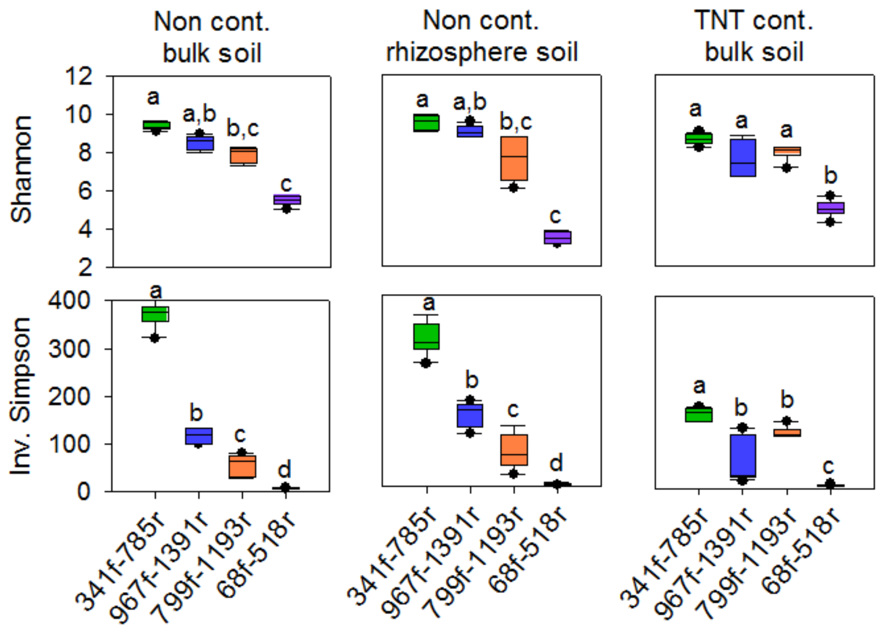

b. Rarefied OTU-table: Shannon diversity and inverse Simpson

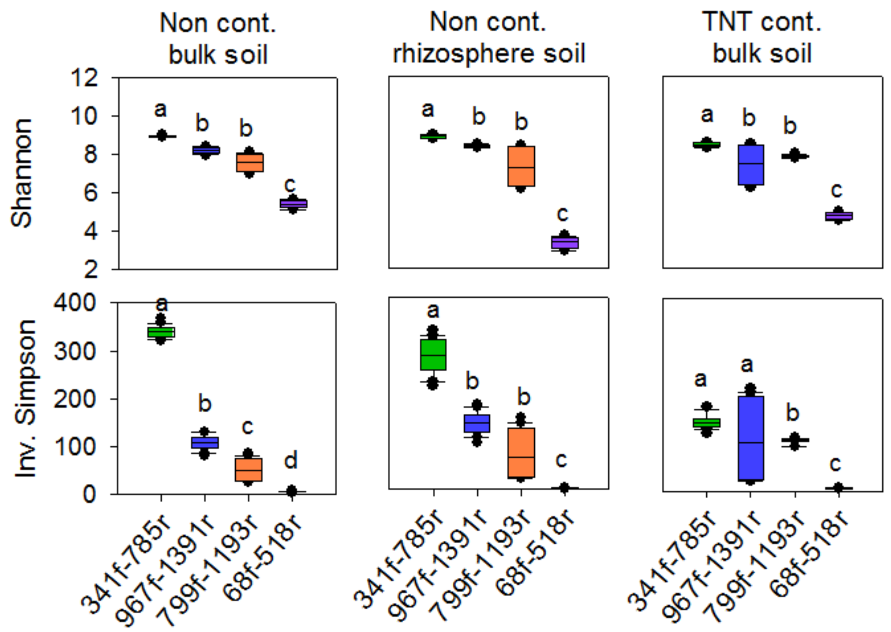

Supplementary Figure 5: **Box plots of Shannon diversity and Inverse Simpson diversity estimates for the four primer pairs used in this study, 68f/518r, 341f/785r, 799f/1193r, 967f/1391r**. Averages were calculated based on the (**a**) non-normalized OTU-table and (**b**) rarefied OTU-table at 1,000 reads. Different letters denote significant differences (Kruskal Wallis, $p < 0.05$).
