# Supplementary material for: Comparative Evaluation of Four Bacteria-Specific Primer Pairs for 16S rRNA Gene Surveys
Source: Front Microbiol. 2017 Mar 28;8:494. doi: 10.3389/fmicb.2017.00494 (PMC5368227; doi:10.3389/fmicb.2017.00494)
Supplement: Supplementary file 11 [file Image6.PDF]

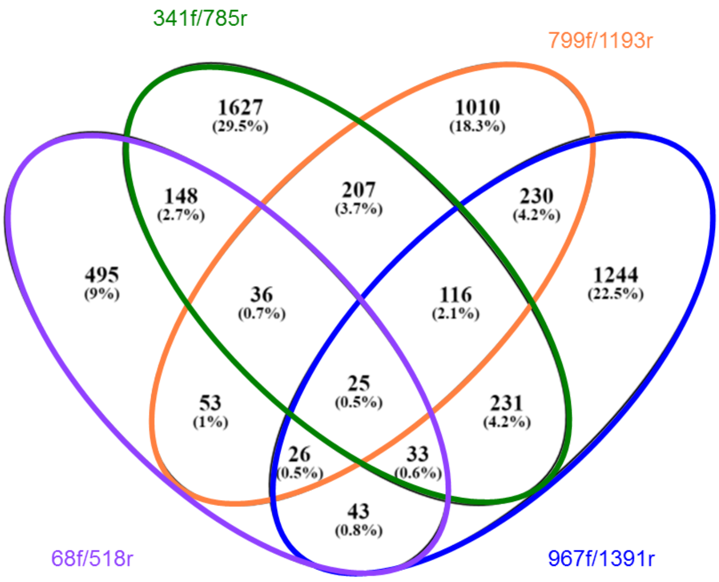

Shared OTUs	341f/785r	799f/1193r	967f/1391r
68f/518r	242 (4.3 %)	140 (2.5 %)	127 (2.3 %)
341f/785r		384 (6.9%)	405 (7.3 %)
799f/1193r			397 (7.1 %)

Supplementary Figure 6: Shared and unique OTUs amongst the four primer pairs used in this study, 68f/518r, 341f/785r, 799f/1193r, 967f/1391r. Average number of observed species was based on 97 % sequence similarity cut-off value.
