# Supplementary material for: Comparative Evaluation of Four Bacteria-Specific Primer Pairs for 16S rRNA Gene Surveys
Source: Front Microbiol. 2017 Mar 28;8:494. doi: 10.3389/fmicb.2017.00494 (PMC5368227; doi:10.3389/fmicb.2017.00494)
Supplement: Supplementary file 13 [file Image8.PDF]

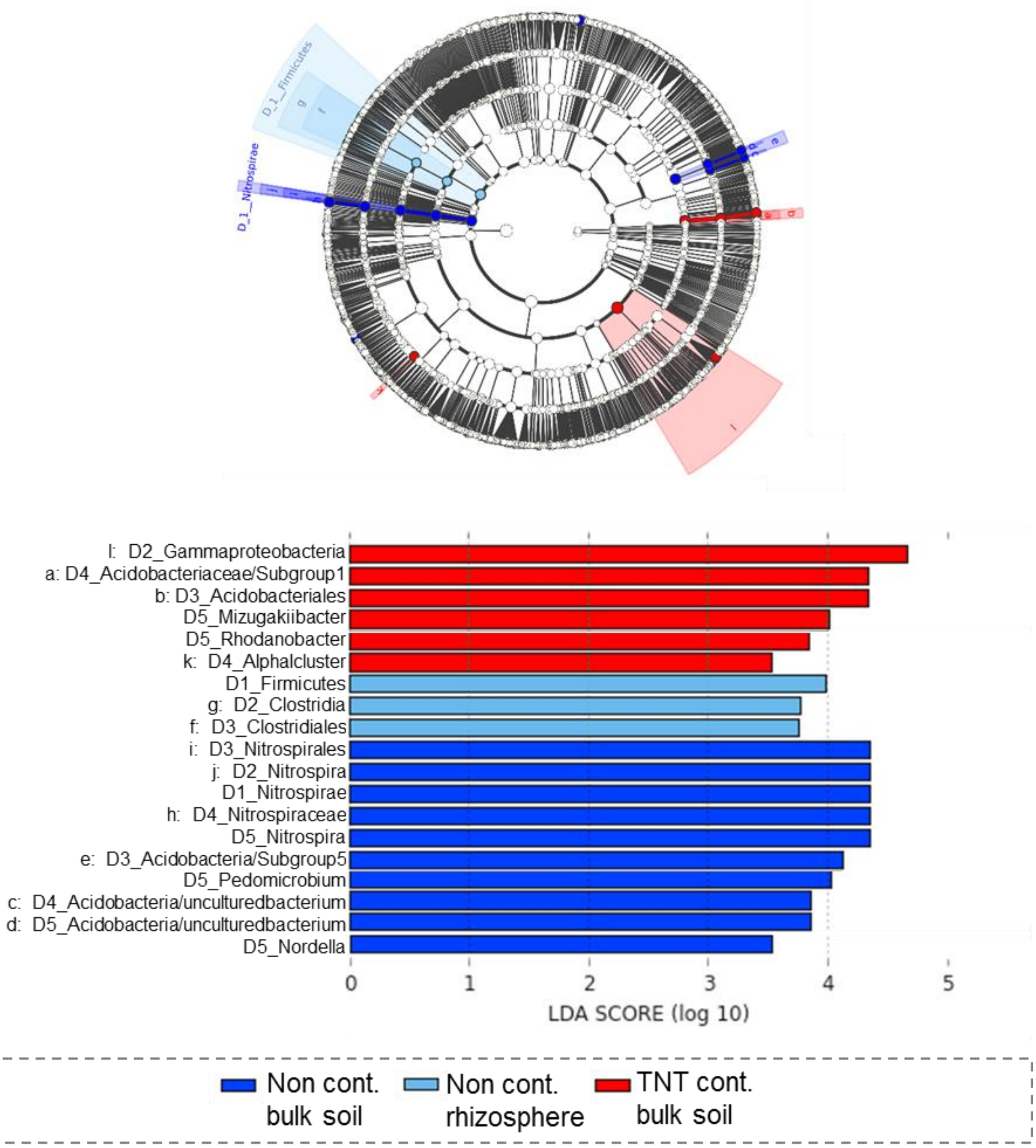

Supplementary Figure 8: Significantly different clades between the soils as determined using LefSe. Only the most significant clades with an LDA-score > 3 are shown.
