# Supplementary material for: Comparative Evaluation of Four Bacteria-Specific Primer Pairs for 16S rRNA Gene Surveys
Source: Front Microbiol. 2017 Mar 28;8:494. doi: 10.3389/fmicb.2017.00494 (PMC5368227; doi:10.3389/fmicb.2017.00494)
Supplement: Supplementary file 14 [file Image9.PDF]

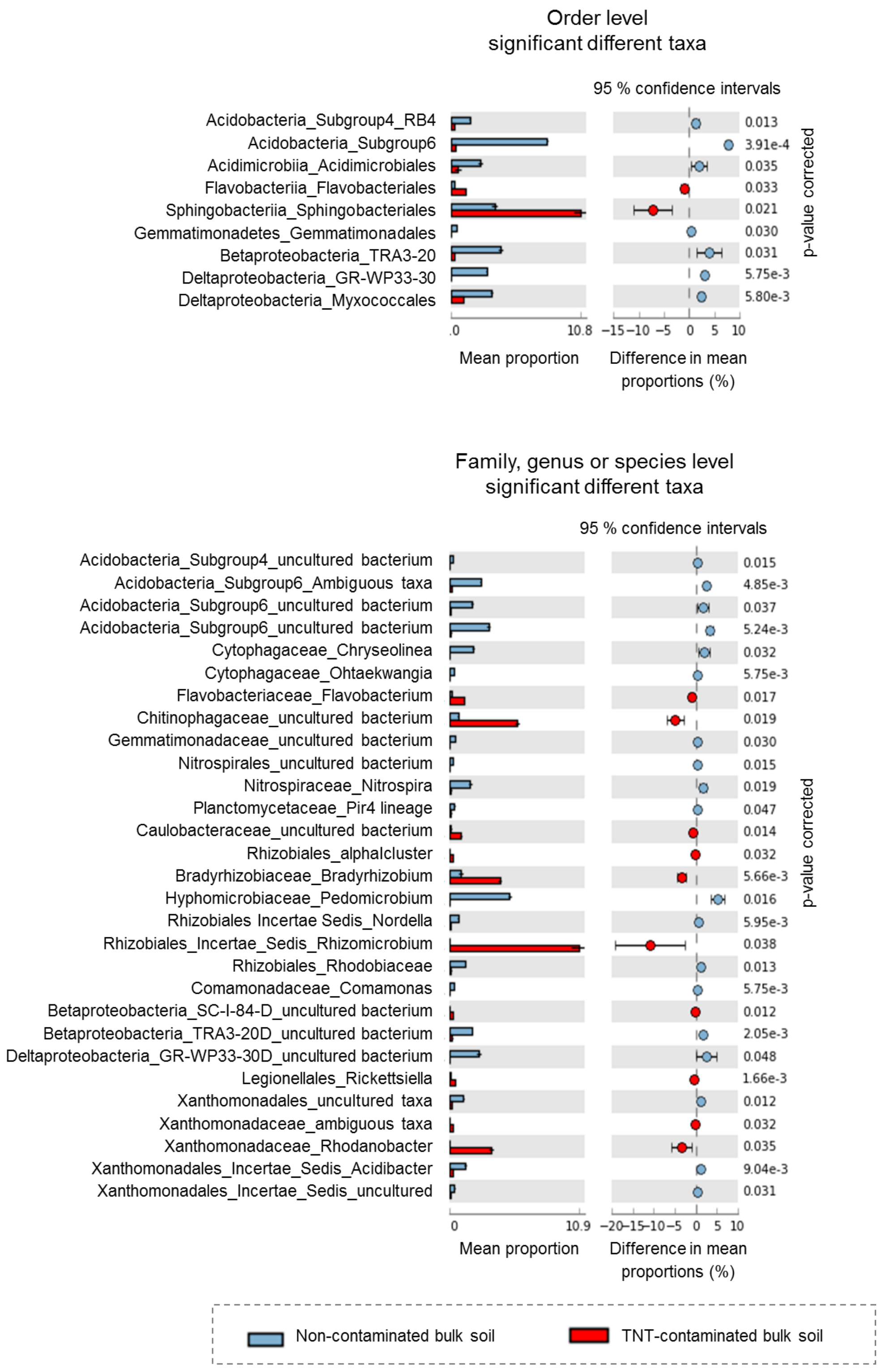

Supplementary Figure 9: Differently enriched taxa in the non-contaminated and TNT-contaminated bulk soil as detected by 341f/785r. Significant taxa were determined using Welch's t-test ($p < 0.05$) in STAMP.
