# Supplementary figures and images for: Comparative Evaluation of Four Bacteria-Specific Primer Pairs for 16S rRNA Gene Surveys
Source: Front Microbiol. 2017 Mar 28;8:494. doi: 10.3389/fmicb.2017.00494 (PMC5368227; doi:10.3389/fmicb.2017.00494)

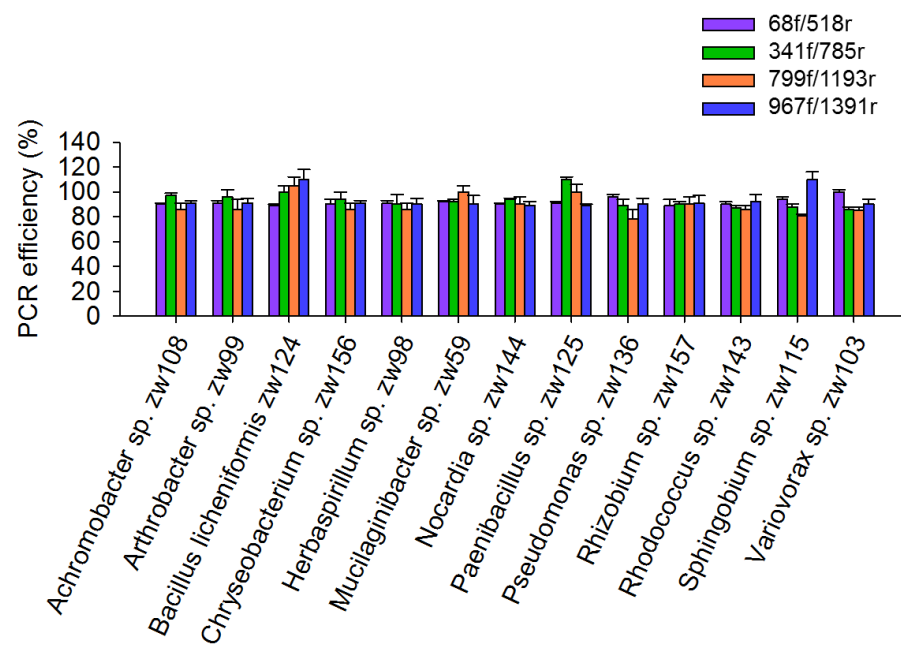

**Supplementary Figure 10: PCR efficiency for single strains.**

Supplement: Supplementary file 15 [file Image10.PDF]
