# Supplementary material for: Comparative Evaluation of Four Bacteria-Specific Primer Pairs for 16S rRNA Gene Surveys
Source: Front Microbiol. 2017 Mar 28;8:494. doi: 10.3389/fmicb.2017.00494 (PMC5368227; doi:10.3389/fmicb.2017.00494)
Supplement: Supplementary file 16 [file Image11.PDF]

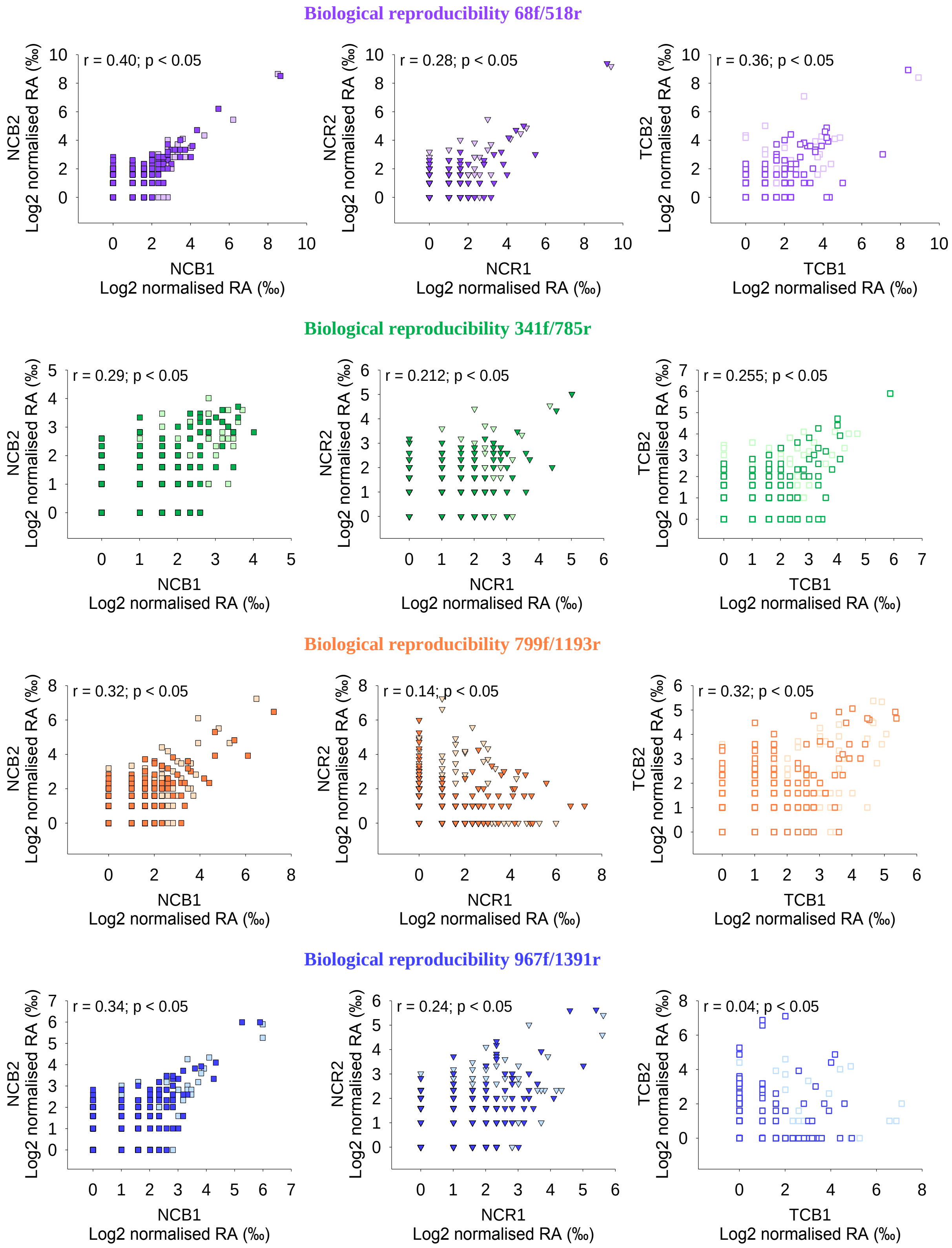

Supplementary Figure 11: Biological reproducibility. NCB is noncontaminated bulk soil, NCR non-contaminated rhizosphere, TCB is TNT-contaminated bulk soil.
