# Supplementary material for: Comparative Evaluation of Four Bacteria-Specific Primer Pairs for 16S rRNA Gene Surveys
Source: Front Microbiol. 2017 Mar 28;8:494. doi: 10.3389/fmicb.2017.00494 (PMC5368227; doi:10.3389/fmicb.2017.00494)
Supplement: Supplementary file 17 [file Image12.PDF]

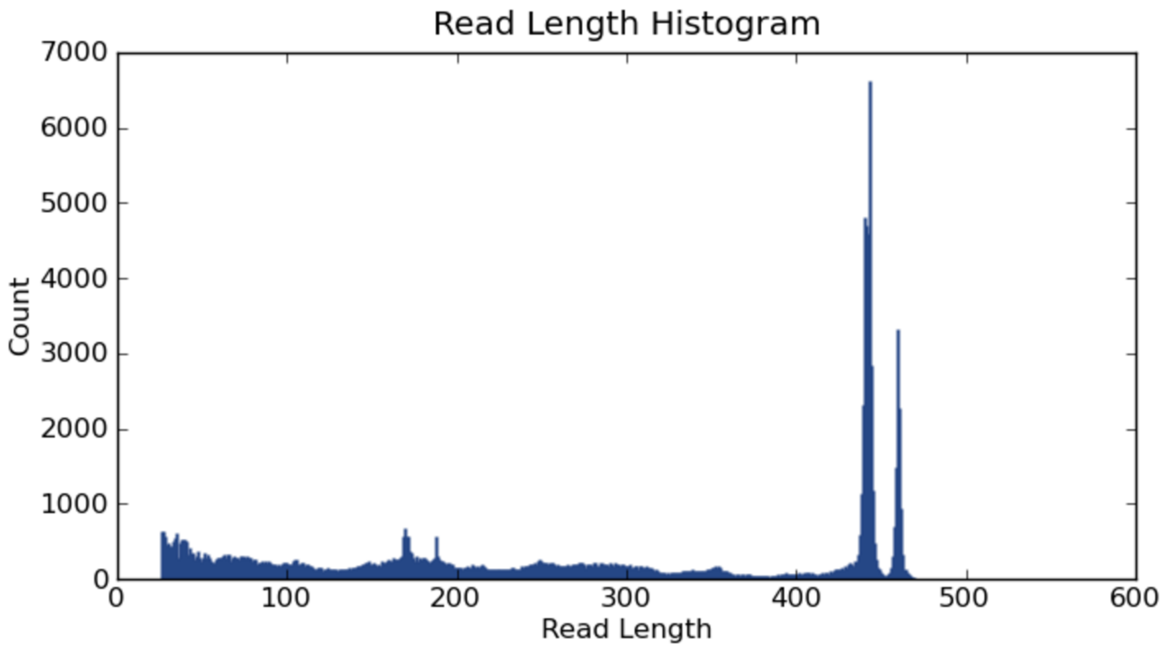

Supplementary Figure 12: Raw 16S rRNA gene sequence length distribution as generated by Ion Torrent sequencing using primer pair 341f/785r.
