# Supplementary material for: Comparative Evaluation of Four Bacteria-Specific Primer Pairs for 16S rRNA Gene Surveys
Source: Front Microbiol. 2017 Mar 28;8:494. doi: 10.3389/fmicb.2017.00494 (PMC5368227; doi:10.3389/fmicb.2017.00494)
Supplement: Supplementary file 18 [file Image13.PDF]

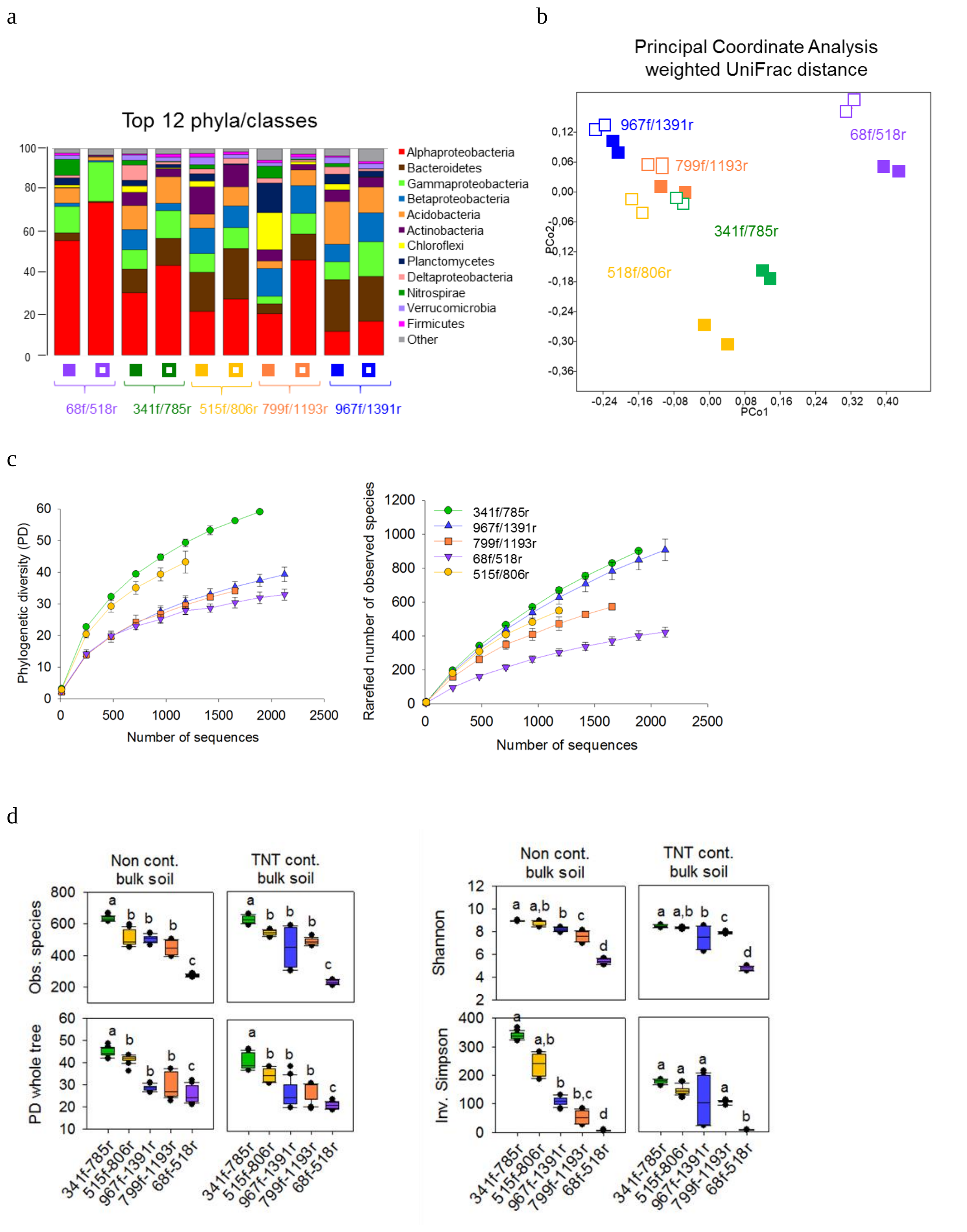

Supplementary Figure 13: Comparative analyses between the four primer pairs tested in this study, 68f/518r, 341f/785r, 799f/1193r, and 967f/1391r, and the bacteria/archaea primer set 515f/806r on the same non-contaminated and contaminated bulk soil samples. a. Twelve most abundant phyla/classes detected in the non-contaminated (solid squares) and explosives contaminated bulk soil soil samples (open squares) from a military forest, Zwijndrecht, Belgium. b. PCoA plot of the CSS-normalised OTU-table and weighted UniFrac distance matrix. C. Rarefaction curves. D. Box plots of observed species, PD whole tree, Shannon diversity and inverse Simpson index based on the rarefied OTU-table. Different letters denote significant differences (Kruskal Wallis, $p < 0.05$).
